# Chloroplast variation is incongruent with classification of the Australian bloodwood eucalypts (genus *Corymbia*, family Myrtaceae)

**DOI:** 10.1371/journal.pone.0195034

**Published:** 2018-04-18

**Authors:** Tanja M. Schuster, Sabrina D. Setaro, Josquin F. G. Tibbits, Erin L. Batty, Rachael M. Fowler, Todd G. B. McLay, Stephen Wilcox, Peter K. Ades, Michael J. Bayly

**Affiliations:** 1 School of BioSciences, The University of Melbourne, Parkville, VIC, Australia; 2 National Herbarium of Victoria, Royal Botanic Gardens Victoria, Birdwood Avenue, South Yarra, VIC, Australia; 3 Department of Biology, Wake Forest University, Winston-Salem, NC,United States of America; 4 Department of Economic Development, Jobs, Transport and Resources, AgriBiosciences Centre, La Trobe University, Bundoora, VIC, Australia; 5 Genomics Hub, The Walter and Eliza Hall Institute of Medical Research, 1G Royal Parade, Parkville, Melbourne, VIC, Australia; 6 School of Ecosystem and Forest Sciences, The University of Melbourne, Parkville, Melbourne, VIC, Australia; National Cheng Kung University, TAIWAN

## Abstract

Previous molecular phylogenetic analyses have resolved the Australian bloodwood eucalypt genus *Corymbia* (~100 species) as either monophyletic or paraphyletic with respect to *Angophora* (9–10 species). Here we assess relationships of *Corymbia* and *Angophora* using a large dataset of chloroplast DNA sequences (121,016 base pairs; from 90 accessions representing 55 *Corymbia* and 8 *Angophora* species, plus 33 accessions of related genera), skimmed from high throughput sequencing of genomic DNA, and compare results with new analyses of nuclear ITS sequences (119 accessions) from previous studies. Maximum likelihood and maximum parsimony analyses of cpDNA resolve well supported trees with most nodes having >95% bootstrap support. These trees strongly reject monophyly of *Corymbia*, its two subgenera (*Corymbia* and *Blakella*), most taxonomic sections (*Abbreviatae*, *Maculatae*, *Naviculares*, *Septentrionales*), and several species. ITS trees weakly indicate paraphyly of *Corymbia* (bootstrap support <50% for maximum likelihood, and 71% for parsimony), but are highly incongruent with the cpDNA analyses, in that they support monophyly of both subgenera and some taxonomic sections of *Corymbia*. The striking incongruence between cpDNA trees and both morphological taxonomy and ITS trees is attributed largely to chloroplast introgression between taxa, because of geographic sharing of chloroplast clades across taxonomic groups. Such introgression has been widely inferred in studies of the related genus *Eucalyptus*. This is the first report of its likely prevalence in *Corymbia* and *Angophora*, but this is consistent with previous morphological inferences of hybridisation between species. Our findings (based on continent-wide sampling) highlight a need for more focussed studies to assess the extent of hybridisation and introgression in the evolutionary history of these genera, and that critical testing of the classification of *Corymbia* and *Angophora* requires additional sequence data from nuclear genomes.

## Introduction

**Fig 1:**
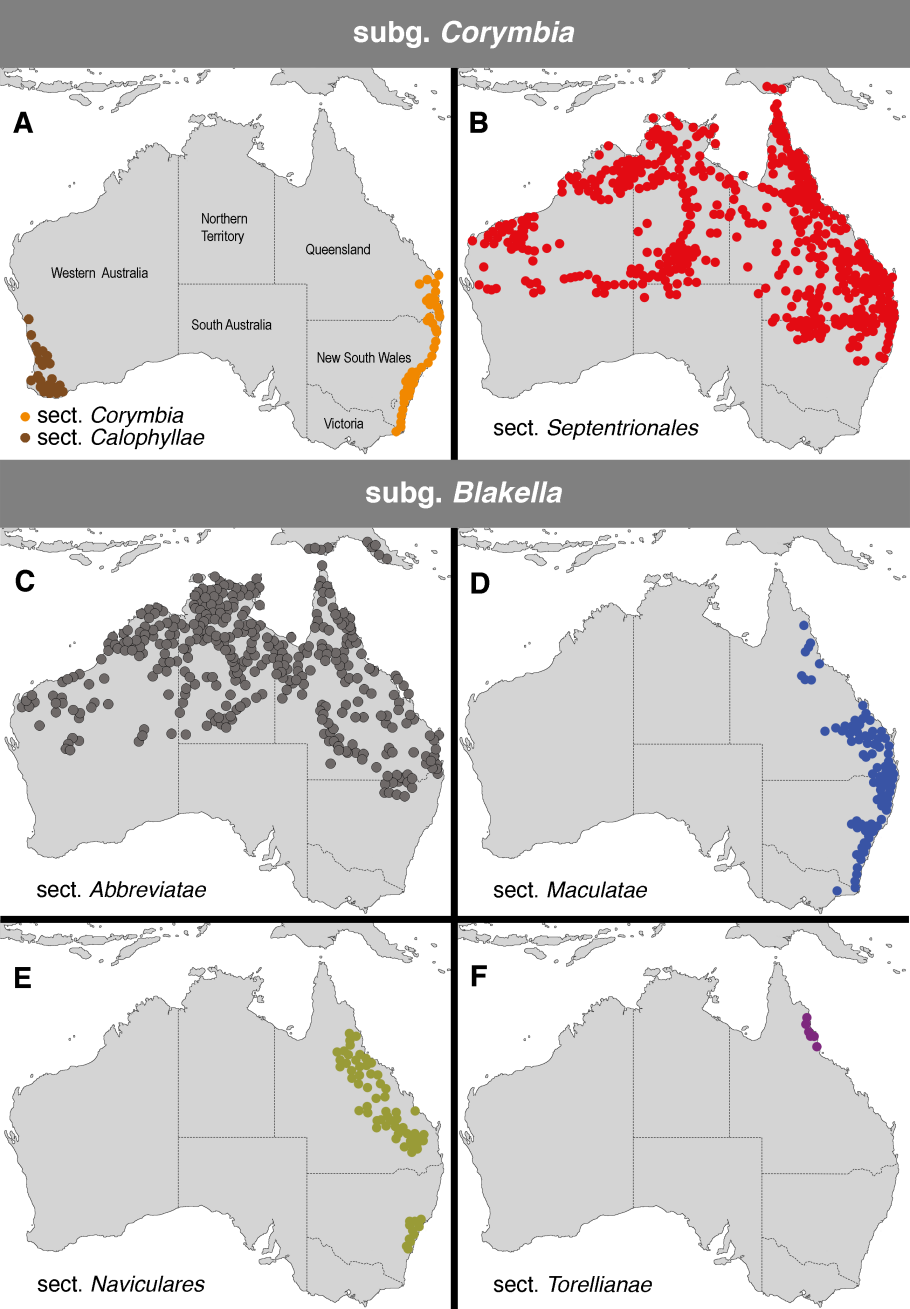
Distribution of infrageneric groups in *Corymbia* (adapted from [2] and following the classification of [6]). Colour coding of groups matches that used in other figures, i.e., taxonomic sections of *Corymbia sensu* Parra-Osorio et al. [6].

**Fig 2:**
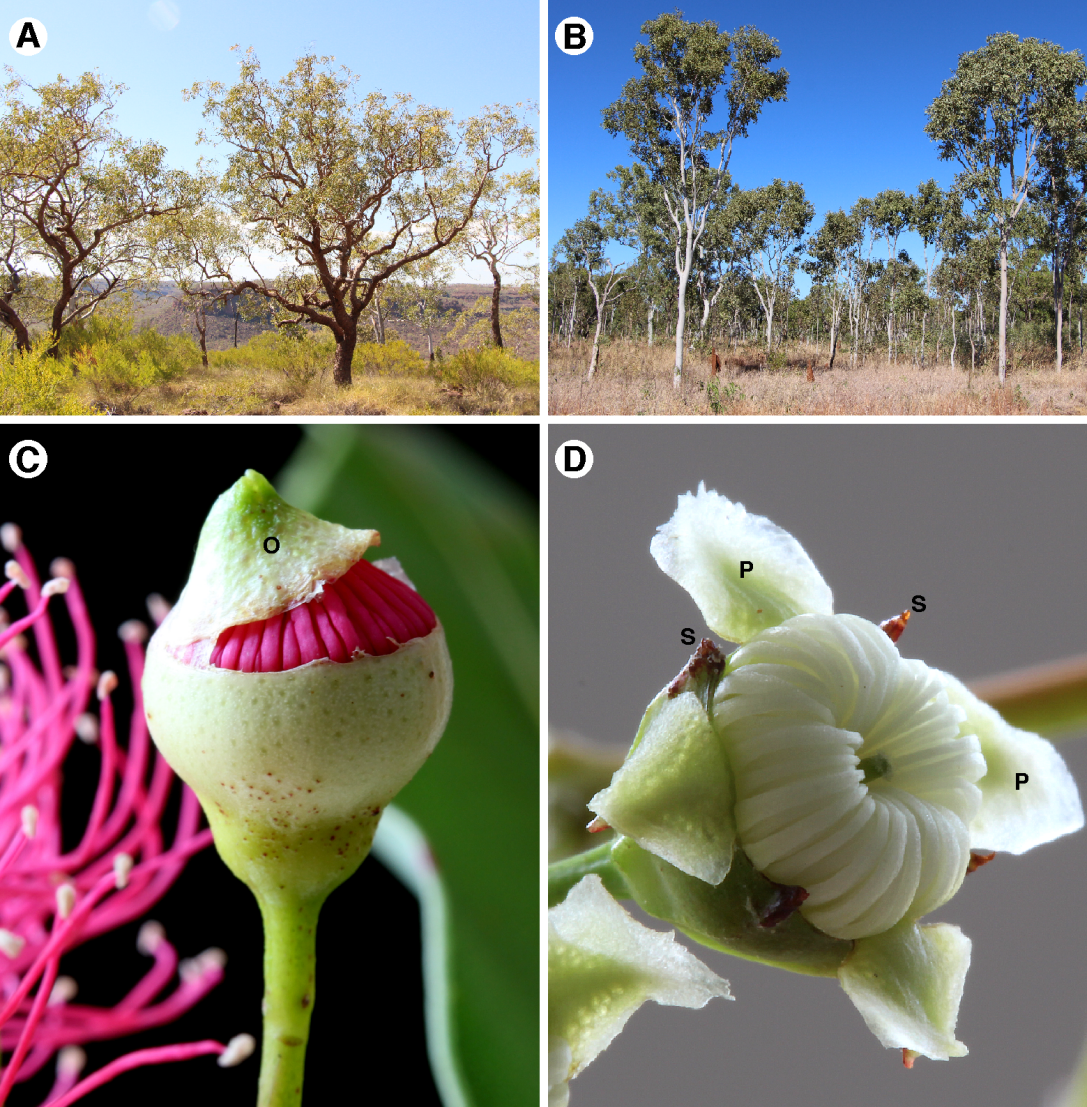
Photographs of *Corymbia* and *Angophora*. (A) Woodland dominated by *Corymbia cliftoniana* (a red bloodwood, sect. *Septentrionales*), near Victoria River, Northern Territory; (B) woodland dominated by *Corymbia grandifolia* (a ghost gum, sect. *Abbreviatae*), near Daly Waters, Northern Territory; (C) partially opened flower bud of *Corymbia ficifolia* (sect. *Calophyllae*), showing operculate perianth (O) separating from hypanthium; (D) open flower of *Angophora floribunda* showing perianth composed of five, free sepals (S) and five, free petals (P).

The bloodwood eucalypts are sclerophyllous trees (c. 100 species) [1], currently classified in the genus *Corymbia* K.D.Hill & L.A.S.Johnson, which was taxonomically segregated from the genus *Eucalyptus* L'Her. in 1995 [2]. Bloodwoods include several morphologically distinct groups that have been formally or informally classified at a range of taxonomic levels (e.g. [2,3,4,5]), and are here identified (following Parra-Osorio et al. [6]) as the red bloodwoods (subg. *Corymbia*), yellow bloodwoods (subg. *Blakella* sect. *Naviculares*), ghost gums or paper-fruited bloodwoods (subg. *Blakella* sect. *Abbreviatae*), spotted gums (subg. *Blakella* sect. *Maculatae*), and cadaghi (monotypic subg. *Blakella* sect. *Torellianae*). These groups occur primarily in northern or eastern Australia (Fig 1), where they are well-represented in monsoonal, tropical savannahs, and there are two small groups of red bloodwoods restricted to the south-west and south-east of Australia (sections *Calophyllae* and *Corymbia*, respectively) in areas with Mediterranean, temperate climates. Bloodwoods are dominant trees in many of the areas in which they occur (Fig 2) and are thus ecologically important [7]; they also have a history of traditional and modern uses, and some species are widely grown for timber, for pulp, or as ornamental trees [7,8,9]. Traditional uses include kino (hardened sap), which is blood-red and confers the groups’ common name, utilised for art and medicinal purposes [10,11,12], and *C*. *citriodora* yields essential oils used as insect repellent and that have antimicrobial and antifungal properties [13,14,15]. *Corymbia* is part of the "eucalypt group" [16] (tribe Eucalypteae) [17] that also includes the sclerophyll genera *Eucalyptus* (>665 species) [1] and *Angophora* (9–10 species) [1,18], and the rainforest genera *Arillastrum* (1 species) [19], *Stockwellia* (1 species) [20], *Allosyncarpia* (1 species) [21], and *Eucalyptopsis* (2 species) [22].

The taxonomic splitting of *Corymbia* from *Eucalyptus* was contentious (e.g. [4,23]), with the key motivation for the separation of *Corymbia* being that the bloodwoods, on the basis of both morphological analyses [2,24] and early molecular analyses [25], were more closely related to *Angophora* than to *Eucalyptus*. That relationship has been unequivocally supported by all subsequent molecular phylogenetic analyses of the group (e.g. [6,26,27,28,29,30,31]), and is supported by some morphological characters, including patterns of leaf venation, features of trichomes, and the presence of oil ducts in the pith of branches [2,24,32]. There are, nonetheless, clear differences in some macro-morphological features between the two groups that have led to the longstanding treatment of *Angophora* as a separate genus from the bloodwoods (whether placed in *Eucalyptus* or treated as *Corymbia*) by almost all authors (e.g., [5,8,9,18,33,34,35]) since *Angophora* was first described in 1797 [36]. The most notable differences between the groups are in the flowers, which in *Angophora* have free sepals and petals, in contrast to the calyptrate/operculate perianth of *Corymbia* (Fig 2). Despite such morphological differences, molecular phylogenetic analyses have presented conflicting signals regarding monophyly of *Corymbia* [37], with some resolving the genus as monophyletic (e.g. [6,27,38,39]), while others resolve it as paraphyletic, with *Angophora* nested within it [28,29,30,31,40].

Most phylogenetic analyses assessing the relationships of bloodwoods to other eucalypts have employed few DNA markers generated by conventional Sanger sequencing methods (e.g. [6,26,27,28,29,30,31]). The use of High-Throughput Sequencing (HTS) methods, which can generate larger volumes of sequence data, are only just beginning to be used in eucalypt studies (e.g. [41]). Partly as a result of the small size of most molecular datasets, some key relationships have typically been poorly supported, including that of the bloodwoods to *Angophora*. For example, Maximum Parsimony (MP) bootstrap support values indicating paraphyly of *Corymbia* have generally been in the range of 51–93% for clades showing *Angophora* nested in *Corymbia* [28,30,40], and those for a monophyletic *Corymbia* have ranged from 78–100% [6,27,38,39]. An exception is a recent study that used analyses of whole chloroplast (cp) genomes [41], which showed strong support for *Corymbia* as paraphyletic with respect to *Angophora* (parsimony bootstrap support and Bayesian posterior probability both 100%), with subg. *Blakella* being more closely related to *Angophora* than to subg. *Corymbia*. However, that study, despite using a large amount of sequence data, included only one sample each of the red bloodwoods, yellow bloodwoods, ghost gums and spotted gums, and it was unclear whether the result was an artefact of sparse taxon sampling.

In this study, we assess relationships of bloodwood eucalypts, including those among species, series, sections and subgenera of *Corymbia*, and those of *Corymbia* to *Angophora*. We expand on the sampling of Bayly et al. [41] to include the largest sample of species of *Angophora* and *Corymbia* in any molecular study using HTS data to date (Table 1). We specifically address questions relating to the evolutionary history of this large and ecologically and economically important group and test the current taxonomic classification, especially at the ranks of genus and subgenus. We use both chloroplast genome derived sequences and combine sequences of nuclear ribosomal internal transcribed spacer (ITS) regions from previous studies for separate phylogenetic analyses using Maximum Likelihood (ML) and MP methods, to provide assessment of phylogenetic signal from both nuclear and chloroplast markers. Specifically these methods are used to test the hypotheses that 1) *Corymbia* is monophyletic and 2) the currently recognised subgenera [6] are monophyletic.

**Table 1:** Details of accessions used in this study.

Genus and species	Subgenus	Section/series	Collector number, herbarium voucher location	Provenance/reference	GenBank accession
**Outgroups**					
*Allosyncarpia ternata* S.T.Blake			Spokevicius A. s.n., MELU 108348	QLD, Sankowsky Arboretum, Tolga [41]	NC_022413.1
*Stockwellia quadrifida* D.J.Carr, S.G.M.Carr & B.Hyland			Spokevicius A. s.n., MELU 108349	QLD, Sankowsky Arboretum, Tolga [41]	NC_022414.1
***Angophora***					
*A*. *bakeri* E.C.Hall			TMS14–27, MEL	NSW, Windsor	KY246359
*A*. *costata* (Gaertn.) Britten			Ades P. s.n., MELU 108350	VIC, UoM, Creswick [41]	NC_022412.1
*A*. *costata* subsp. *costata*			DN2073, AD 163314	NSW, Glenbrook	KY246360
*A*. *costata* subsp. *euryphylla* L.A.S.Johnson ex G.J.Leach			DN2082 ^i^, AD 163326	NSW, Broken Back Range	KY246361
*A*. *costata* subsp. *euryphylla* L.A.S.Johnson ex G.J.Leach			TMS14–29, MEL	NSW, Yengo National Park	KY246362
*A*. *floribunda* (Sm.) Sweet			Spokevicius A. s.n., MELU 108351	VIC, UoM, Creswick [41]	NC_022411.1
*A*. *floribunda*			MJB2471, MELU 114352	VIC, Mallacoota	KY246363
*A*. *floribunda*			TMS14–33, MEL	NSW, Ulan	KY246364
*A*. *hispida* (Sm.) Blaxell			KLW11152, NSW	NSW, Royal National Park	KY246365
*A*. *inopina* K.D.Hill			DN6258, AD	NSW, Wyee	KY246366
*A*. *leiocarpa* (L.A.S.Johnson ex G.J.Leach) K.R.Thiele & Ladiges			DN2103, AD 163292	QLD, Karara	KY246367
*A*. *melanoxylon* R.T.Baker			CAPO–R173–P1, MELU 107825	QLD, St George	KY246368
*A*. *melanoxylon*			DN2552, AD 163739	QLD, St George	KY246369
*A*. *subvelutina* F.Muell.			TMS14–28, MEL	NSW, Windsor	KY246370
*A*. *subvelutina*			DN3204, AD, NSW	NSW, Mulgoa	KY246371
***Corymbia***					
*C*. *abergiana* (F.Muell.) K.D.Hill & L.A.S.Johnson	*Corymbia*	*Septentrionales/ Abergianae*	CAPO23–891966, MELU 107776	NSW, Mount Annan BG, cultivated	KY246372
*C*. *aparrerinja* K.D.Hill & L.A.S.Johnson	*Blakella*	*Abbreviatae/ Grandifoliae*	MJB2485, MELU 114286	NT, 108.4 km N of Alice Springs on Stuart Highway	KY246373
*C*. *aparrerinja*	*Blakella*	*Abbreviatae/ Grandifoliae*	MJB2494, MELU 114292	NT, 33 km N of Devils Marbles, Stuart Highway	KY246374
*C*. *arafurica* K.D.Hill & L.A.S.Johnson	*Blakella*	*Abbreviatae/ Papuanae*	MJB2553, MELU 114349	NT, 32.2 km E Humpty Doo, Arnhem Highway	KY246375
*C*. *arafurica*	*Blakella*	*Abbreviatae/ Papuanae*	MJB2555, MELU 114351	NT, 5.8 km E Bark Hut roadhouse, Arnhem Highway	KY246376
*C*. *arnhemensis* (D.J.Carr & S.G.M.Carr) K.D.Hill & L.A.S.Johnson	*Corymbia*	*Septentrionales/ Arenariae*	MJB2534, MELU 114330	NT, Nitmiluk National Park	KY246377
*C*. *arnhemensis*	*Corymbia*	*Septentrionales/ Arenariae*	MJB2537, MELU 114333	NT, Nitmiluk National Park	KY246378
*C*. *aspera* (F.Muell.) K.D.Hill & L.A.S.Johnson	*Blakella*	*Abbreviatae/Asperae*	MJB2501, MELU 114300	NT, Stuart Highway, turnoff to Helen Springs	KY246379
*C*. *aspera*	*Blakella*	*Abbreviatae/Asperae*	MJB2503, MELU 114302	NT, 16.7 km N Renner Springs, Stuart Highway	KY246380
*C*. *aureola* (Brooker & A.R.Bean) K.D.Hill & L.A.S.Johnson	*Blakella*	*Naviculares*	DN2542, AD 163675	QLD, Cherwell Range	KY246381
*C*. *bella* K.D.Hill & L.A.S.Johnson	*Blakella*	*Abbreviatae/ Papuanae*	DN4204, AD 164468	NT, Bulman	KY246382
*C*. *bella*	*Blakella*	*Abbreviatae/ Papuanae*	MJB2545, MELU 114341	NT, 10 km N Hayes Creek, Dorat Road	KY246383
*C*. *bella*	*Blakella*	*Abbreviatae/ Papuanae*	MJB2548, MELU 114344	NT, 25.6 km N Adelaide River, Stuart Highway	KY246384
*C*. *bleeseri* (Blakely) K.D.Hill & L.A.S.Johnson	*Corymbia*	*Septentrionales/ Collinae*	MJB2518, MELU 114315	NT, 54 km N Mataranka, Stuart Highway	KY246385
*C*. *bloxsomei* (Maiden) K.D.Hill & L.A.S.Johnson	*Blakella*	*Naviculares*	DN687, AD 162445	QLD, Barakula State Forest	KY246386
*C*. *brachycarpa* (D.J.Carr & S.G.M.Carr) K.D.Hill & L.A.S.Johnson	*Corymbia*	*Septentrionales/ Rhodopes*	CAPO8–891880, MELU 107775	NSW, Mount Annan BG, cultivated	KY246387
*C*. *bunites* (Brooker & A.R.Bean) K.D.Hill & L.A.S.Johnson	*Blakella*	*Naviculares*	DN711, AD 162630	QLD, Blackdown Tableland	KY246388
*C*. *calophylla* (Lindl.) K.D.Hill & L.A.S.Johnson	*Corymbia*	*Calophyllae*	DN1160, AD 99625330	WA, Cockleshell Gully	KY246389
*C*. *calophylla*	*Corymbia*	*Calophyllae*	KLW11208, NSW 972973	WA, 18.6 km NE of Jurien on road to Brand Highway	KY246390
*C*. *calophylla*	*Corymbia*	*Calophyllae*	DN3421, PERTH 5788722	WA, Brookton	KY246391
*C*. *chippendalei* (D.J.Carr & S.G.M.Carr) K.D.Hill & L.A.S.Johnson	*Corymbia*	*Septentrionales*/*Dichromophloiae*	NG6497, MEL 2390093	WA, Katjarra	KY246393
*C*. *citriodora* (Hook.) K.D.Hill & L.A.S.Johnson	*Blakella*	*Maculatae*	DN702, AD 162591	QLD, Springsure	KY246394
*C*. *citriodora*	*Blakella*	*Maculatae*	DN1246 ^ii^, AD 99624278	NSW, Drake	KY246442
*C*. *clarksoniana* (D.J.Carr & S.G.M.Carr) K.D.Hill & L.A.S.Johnson	*Corymbia*	*Septentrionales/ Polycarpae*	CAPO1A–904971, MELU 107760	NSW, Mount Annan BG, cultivated	KY246395
*C*. *collina* (W.Fitzg.) K.D.Hill & L.A.S.Johnson	*Corymbia*	*Septentrionales/ Collinae*	Ladiges P. s.n. MELU	WA, Kimberly region	KY246396
*C*. *deserticola* (D.J.Carr & S.G.M.Carr) K.D.Hill & L.A.S.Johnson subsp. *deserticola*	*Corymbia*	*Septentrionales/ Ferrugineae*	DN542, PERTH 5665884	WA, Weelarrana, Kumarina Roadhouse	KY246397
*C*. *dichromophloia* (F.Muell.) K.D.Hill & L.A.S.Johnson	*Corymbia*	*Septentrionales*/*Dichromophloiae*	MJB2500A, MELU 114298	NT, 28 km N Three Ways, Stuart Highway	KY246398
*C*. *dichromophloia*	*Corymbia*	*Septentrionales*/*Dichromophloiae*	DN4192 ^iii^, AD 164321	NT, Barkly Tableland	KY246392
*C*. *dichromophloia*	*Corymbia*	*Septentrionales/ Dichromophloiae*	DN1928 ^iv^, PERTH 5218063	WA, Gibb River Road	KY246434
*C*. *dunlopiana* K.D.Hill & L.A.S.Johnson	*Corymbia*	*Septentrionales/ Ferrugineae*	MJB2533, MELU 114329	NT, 14.1. km E Katherine, Katherine Gorge Road	KY246399
*C*. *ellipsoidea* (D.J.Carr & S.G.M.Carr) K.D.Hill & L.A.S.Johnson	*Corymbia*	*Septentrionales*/*Dichromophloiae*	DN5112, AD	QLD, Mount Garnet	KY246400
*C*. *eremaea* subsp. *oligocarpa* (Blakely & Jacobs) K.D.Hill & L.A.S.Johnson	*Corymbia*	*Septentrionales*/*Dichromophloiae*	DN1208, AD 99625502	NT, Mount Gillen	KY246401
*C*. *eximia* (Schauer) K.D.Hill & L.A.S.Johnson	*Blakella*	*Naviculares*	DN632, AD 162956	NSW, Hawksbury River scarp near Wisemans Ferry	KY246402
*C*. *eximia*	*Blakella*	*Naviculares*	MJB2237, MELU 108352	VIC, PFP Arboretum [41]	NC_022409.1
*C*. *ferriticola* (Brooker & Edgecombe) K.D.Hill & L.A.S.Johnson	*Blakella*	*Abbreviatae/Asperae*	DN2752, AD 163641	WA, Meekatharra	KY246403
*C*. *ferruginea* (Schauer) K.D.Hill & L.A.S.Johnson	*Corymbia*	*Septentrionales/ Ferrugineae*	MJB2505, MELU 114304	NT, 70.5 km N Renner Springs, Stuart Highway	KY246404
*C*. *ferruginea*	*Corymbia*	*Septentrionales/ Ferrugineae*	MJB2508, MELU 114307	NT, 12.9 km N Dunmarra, Stuart Highway	KY246405
*C*. *ficifolia* (F.Muell.) K.D.Hill & L.A.S.Johnson	*Corymbia*	*Calophyllae*	KLW11249, NSW 973354	WA, Walpole, Nornalup National Park	KY246406
*C*. *flavescens* K.D.Hill & L.A.S.Johnson	*Blakella*	*Abbreviatae/ Grandifoliae*	MJB2498, MELU 114296	NT, 18 km N Three Ways, Stuart Highway	KY246407
*C*. *foelscheana* (F.Muell.) K.D.Hill & L.A.S.Johnson	*Corymbia*	*Septentrionales*/*Dichromophloiae*	MJB2540, MELU 114336	NT, Nitmiluk National Park, track to Edith Falls	KY246408
*C*. *grandifolia* (R.Br. ex Benth.) K.D.Hill & L.A.S.Johnson	*Blakella*	*Abbreviatae/ Grandifoliae*	MJB2511, MELU 114310	NT, 4.6 km E Daly Waters, Carpentaria Highway	KY246409
*C*. *grandifolia*	*Blakella*	*Abbreviatae/ Grandifoliae*	MJB2517, MELU 114314	NT, 7.1 km S of turnoff to Forrest Hill Station	KY246410
*C*. *gummifera* (Gaertn.) K.D.Hill & L.A.S.Johnson	*Corymbia*	*Corymbia*	DN1228, AD 99624288	NSW, Ku-Ring-Gai Chase	KY246411
*C*. *gummifera*	*Corymbia*	*Corymbia*	MJB2472, MELU 114353	VIC, Mallacoota	KY246412
*C*. *gummifera*	*Corymbia*	*Corymbia*	MJB2232, MELU 108355	VIC, PFP Arboretum [41]	NC_022407.1
*C*. *haematoxylon* (Maiden) K.D.Hill & L.A.S.Johnson	*Corymbia*	*Calophyllae*	KLW11229, NSW 973332	WA, Dardanup Conservation Park	KY246413
*C*. *hamersleyana* (D.J.Carr & S.G.M.Carr) K.D.Hill & L.A.S.Johnson	*Corymbia*	*Septentrionales*/*Dichromophloiae*	DN1190, AD 99625487	WA, Mount Nameless	KY246414
*C*. *hamersleyana*	*Corymbia*	*Septentrionales*/*Dichromophloiae*	CAPO10–832169 ^v^, MELU	NSW, Mount Annan BG, cultivated	KY246435
*C*. *henryi* (S.T.Blake) K.D.Hill & L.A.S.Johnson	*Blakella*	*Maculatae*	DN1259, AD 99624267	QLD, Helidon Hills	KY246415
*C*. *henryi*	*Blakella*	*Maculatae*	DN2092, AD 163302	NSW, Coast Range	KY246416
*C*. *intermedia* (R.T.Baker) K.D.Hill & L.A.S.Johnson	*Corymbia*	*Septentrionales/ Intermediae*	DN708, AD 162627	QLD, Blackdown Tableland	KY246417
*C*. *intermedia*	*Corymbia*	*Septentrionales/ Intermediae*	GB330, MELU	NSW, Nambucca Heads, Black Rock	KY246418
*C*. *jacobsiana* (Blakely) K.D.Hill & L.A.S.Johnson	*Corymbia*	*Septentrionales*	MJB2543B, MELU 114339	NT, Mesa, N side Stuart Highway	KY246419
*C*. *leichhardtii* (F.M.Bailey) K.D.Hill & L.A.S.Johnson	*Blakella*	*Naviculares*	DN1277, AD 99624246	QLD, Springsure	KY246420
*C*. *lenziana* (D.J.Carr & S.G.M.Carr) K.D.Hill & L.A.S.Johnson	*Corymbia*	*Septentrionales*/*Dichromophloiae*	DN5733, AD 241349	WA, Kennedy Range	KY246421
*C*. *ligans* K.D.Hill & L.A.S.Johnson	*Corymbia*	*Septentrionales/ Polycarpae*	DN2527, AD 163755	QLD, Greenvale	KY246422
*C*. *maculata* (Hook.) K.D.Hill & L.A.S.Johnson	*Blakella*	*Maculatae*	DN1752, AD 99623282	NSW, Narooma	KY246423
*C*. *maculata*	*Blakella*	*Maculatae*	Spokevicius A. s.n., MELU 108353	VIC, UoM, Creswick [41]	NC_022408.1
*C*. *opaca* (D.J.Carr & S.G.M.Carr) K.D.Hill & L.A.S.Johnson	*Corymbia*	*Septentrionales*/*Dichromophloiae*	NG6494, MEL 2390090	WA, Katjarra	KY246424
*C*. *pachycarpa* K.D.Hill & L.A.S.Johnson	*Corymbia*	*Septentrionales/ Ferrugineae*	DN1939, PERTH 5218012	WA, Billiluna	KY246425
*C*. *peltata* (Benth.) K.D.Hill & L.A.S.Johnson	*Blakella*	*Naviculares*	DN2505, AD 163709	QLD, Newcastle Range	KY246426
*C*. *petalophylla* (Brooker & A.R.Bean) K.D.Hill & L.A.S.Johnson	*Blakella*	*Naviculares*	DN1262, AD 99624268	QLD, Eidsvold	KY246427
*C*. *plena* K.D.Hill & L.A.S.Johnson	*Corymbia*	*Septentrionales/ Polycarpae*	DN2535, AD 163670	QLD, Torrens Creek	KY246428
*C*. *polysciada* (F.Muell.) K.D.Hill & L.A.S.Johnson	*Blakella*	*Abbreviatae/ Polysciadae*	MJB2544, MELU 114340	NT, 10 km N Hayes Creek, Dorat Road	KY246429
*C*. *polysciada*	*Blakella*	*Abbreviatae/ Polysciadae*	MJB2554, MELU 114350	NT, 300 m E Bark Hut roadhouse, Arnhem Highway	KY246430
*C*. *ptychocarpa* (F.Muell.) K.D.Hill & L.A.S.Johnson	*Corymbia*	*Septentrionales/ Ptychocarpae*	MJB2546A, MELU 114342	NT, Robin Falls, SW Adelaide River	KY246431
*C*. *ptychocarpa*	*Corymbia*	*Septentrionales/ Ptychocarpae*	MJB2551, MELU 114347	NT, Litchfield National Park	KY246432
*C*. *rhodops* (D.J.Carr & S.G.M.Carr) K.D.Hill & L.A.S.Johnson	*Corymbia*	*Septentrionales/ Rhodopes*	DN1306, AD 99624219	QLD, Herberton	KY246433
*C*. *serendipita* (Brooker & Kleinig) Bean	*Corymbia*	*Septentrionales/ Arenariae*	DN2502, AD 163706	QLD, Newcastle Range	KY246436
*C*. *tessellaris* (F.Muell.) K.D.Hill & L.A.S.Johnson	*Blakella*	*Abbreviatae/ Tessellares*	MJB2226, MELU 108354	VIC, PFP Arboretum [41]	NC_022410.1
*C*. *torelliana* (F.Muell.) K.D.Hill & L.A.S.Johnson	*Blakella*	*Torellianae*	TMS14–40, MEL	NSW, Gwydir Highway, Nancy Coulton Lookout, cultivated	KY246437
*C*. *trachyphloia* (F.Muell.) K.D.Hill & L.A.S.Johnson	*Corymbia*	*Septentrionales*	DN684 ^vi^, AD 163256	QLD, Tara	KY246438
*C*. *trachyphloia*	*Corymbia*	*Septentrionales*	DN714, AD 162803	QLD, Goodwood	KY246439
*C*. *trachyphloia*	*Corymbia*	*Septentrionales*	DN5128, AD	QLD, Blencoe Falls	KY246440
*C*. *umbonata* (D.J.Carr & S.G.M.Carr) K.D.Hill & L.A.S.Johnson	*Corymbia*	*Septentrionales/ Dichromophloiae*	MJB2513, MELU 114312	NT, 74 km N Daly Waters, Stuart Highway	KY246441
***Eucalyptus***					
*E*. *aromaphloia* L.D.Pryor & J.H.Willis	*Symphyomyrtus*	*Maidenaria/Acaciiformes*	Ades P. s.n., MELU 108368	VIC, UoM, Creswick [41]	NC_022396.1
*E*. *baxteri* (Benth.) Maiden & Blakely ex J.M.Black	*Eucalyptus*	*Capillulus*	MJB2223, MELU 108360	VIC, PFP Arboretum [41]	NC_022382.1
*E*. *camaldulensis* Dehnh.	*Symphyomyrtus*	*Exsertaria/Rostratae*	Tibbits J. s.n., MELU 108435	VIC, La Trobe University campus, Bundoora [41]	NC_022398.1
*E*. *cladocalyx* F.Muell.	*Symphyomyrtus*	*Sejunctae*	Ades P. s.n., MELU 108383	VIC, UoM, Parkville [41]	NC_022394.1
E. *cloeziana* F.Muell.	*Idiogenes*	*-*	MJB2230, MELU 108357	VIC, PFP Arboretum [41]	NC_022388.1
*E*. *curtisii* Blakely & C.T.White	*Acerosae*	*-*	Spokevicius A. s.n., MELU 108358	VIC, PFP Arboretum [41]	NC_022391.1
*E*. *deglupta* Blume	*Minutifructus*	*Equatoria*	Udovicic F. s.n., MELU 108436	VIC, RBGV, Melbourne [41]	NC_022399.1
*E*. *delegatensis* R.T.Baker	*Eucalyptus*	*Cineraceae*	MJB2134, MELU 108361	VIC, Mt Macedon [41]	NC_022380.1
*E*. *diversicolor* F.Muell.	*Symphyomyrtus*	*Inclusae*	Spokevicius A. s.n., MELU 108373	VIC, PFP Arboretum [41]	NC_022402.1
*E*. *diversifolia* Bonpl.	*Eucalyptus*	*Longistylus*	MJB2229, MELU 108362	VIC, PFP Arboretum [41]	NC_022383.1
*E*. *elata* Dehnh.	*Eucalyptus*	*Aromatica*	Ades P. s.n., MELU 108363	VIC, UoM, Creswick [41]	NC_022385.1
*E*. *erythrocorys* F.Muell.	*Eudesmia*	*Limbatae*	MJB2234, MELU 108356	VIC, PFP Arboretum [41]	NC_022406.1
*E*. *globulus* Labill.	*Symphyomyrtus*	*Maidenaria/Globulares*	Anon. s.n., HO 528199	TAS [42]	NC_008115.1
*E*. *grandis* W.Hill	*Symphyomyrtus*	*Latoangulatae/Transversae*	-	Brazil, Federal University of Viçosa [43]	NC_014570.1
*E*. *guilfoylei* Maiden	*Cruciformes*	*-*	Spokevicius A. s.n., MELU 108359	VIC, PFP Arboretum [41]	NC_022405.1
*E*. *marginata* D.Don ex Sm.	*Eucalyptus*	*Longistylus*	Spokevicius A. s.n., MELU 108369	VIC, PFP Arboretum [41]	NC_022390.1
*E*. *melliodora* A.Cunn. ex Schauer	*Symphyomyrtus*	*Adnataria/Melliodorae*	Spokevicius A. s.n., MELU 108380	VIC, UoM, Creswick [41]	NC_022392.1
*E*. *microcorys* F.Muell.	*Alveolata*	*-*	Spokevicius A. s.n., MELU 108375	VIC, UoM, Creswick [41]	NC_022404.1
*E*. *nitens* (H.Deane & Maiden) Maiden	*Symphyomyrtus*	*Maidenaria/Globulares*	Spokevicius A. s.n., MELU 108378	VIC, UoM, Creswick [41]	NC_022395.1
*E*. *obliqua* L’Hér.	*Eucalyptus*	*Eucalyptus/Eucalyptus*	Spokevicius A. s.n., MELU 108364	VIC, UoM, Creswick [41]	NC_022378.1
*E*. *patens* Benth.	*Eucalyptus*	*Longistylus/Patentes*	MJB2227, MELU 108370	VIC, PFP Arboretum [41]	NC_022389.1
*E*. *polybractea* R.T.Baker	*Symphyomyrtus*	*Adnataria/Buxeales*	Spokevicius A. s.n., MELU 108384	VIC, UoM, Parkville [41]	NC_022393.1
*E*. *radiata* Sieber ex DC.	*Eucalyptus*	*Aromatica/Radiatae*	Ades P. s.n., MELU 108365	VIC, UoM, Creswick [41]	NC_022379.1
*E*. *regnans* F.Muell.	*Eucalyptus*	*Eucalyptus/Regnantes*	MJB2224, MELU 108371	VIC, PFP Arboretum [41]	NC_022386.1
*E*. *saligna* Sm.	*Symphyomyrtus*	*Latoangulatae/Transversae*	Spokevicius A. s.n., MELU 108376	VIC, UoM, Creswick [41]	NC_022397.1
*E*. *salmonophloia* F.Muell.	*Symphyomyrtus*	*Bisectae*/*Salmonophloiae*	MJB2236, MELU 108374	VIC, PFP Arboretum [41]	NC_022403.1
*E*. *sieberi* L.A.S.Johnson	*Eucalyptus*	*Cineraceae*/*Psathyroxylon*	Ades P. s.n., MELU 108366	VIC, UoM, Creswick [41]	NC_022384.1
*E*. *spathulata* Hook.	*Symphyomyrtus*	*Bisectae/Erectae*	Ades P. s.n., MELU 108381	VIC, UoM, Creswick [41]	NC_022400.1
*E*. *torquata* Luehm.	*Symphyomyrtus*	*Dumaria/Torquatae*	Tibbits J. s.n., MELU 108382	VIC, UoM, Creswick [41]	NC_022401.1
*E*. *umbra* R.T.Baker	*Eucalyptus*	*Amentum*	MJB2225, MELU 108372	VIC, PFP Arboretum [41]	NC_022387.1
*E*. *verrucata* Ladiges & Whiffin	*Eucalyptus*	*Capillulus/Pachyphloius*	Tibbits J. s.n., MELU 108367	VIC, UoM, Creswick [41]	NC_022381.1

Classification of subgenera and sections follows [6]; classification of series follows the informal classification of [2], where consistent with the higher-level groupings. CAPO = Carlos A. Parra-Osorio, DN = Dean Nicolle, GB = Gillian Brown, KLW = Karen L. Wilson, MJB = Michael J. Bayly, NG = Neil Gibson, TMS = Tanja M. Schuster. Herbarium codes follow Index Herbariorum: AD = State Herbarium of South Australia, HO = Tasmanian Herbarium, MEL = National Herbarium of Victoria, MELU = University of Melbourne Herbarium, NSW = National Herbarium of New South Wales, PERTH = Western Australian Herbarium. Provenance data: BG = botanic garden, NSW = New South Wales (Australia), NT = Northern Territory (Australia), QLD = Queensland (Australia), TAS = Tasmania (Australia), VIC = Victoria (Australia), WA = Western Australia (Australia). Further abbreviations are PFP = Peter Francis Points Arboretum, Coleraine, Victoria; RBGV = Royal Botanic Garden Victoria, and UoM = The University of Melbourne (, followed by the respective campus).

^i^ Collected as *A*. *euryphylla* (G.J.Leach) L.A.S.Johnson & K.D.Hill, a name that is synonymised with *A*. *costata* subsp. *euryphylla* in the current Australian Plant Census (APC) [1].

^ii^ Collected as *Corymbia variegata* (F.Muell.) K.D.Hill & L.A.S.Johnson, a name that is synonymised with *C*. *citriodora* in the current APC.

^iii^ Collected as *Corymbia capricornia* (D.J.Carr & S.G.M.Carr) K.D.Hill & L.A.S.Johnson, a name that is synonymised with *C*. *dichromophloia* in the current APC.

^iv^ Collected as *Corymbia rubens* K.D.Hill & L.A.S.Johnson, a name that is synonymised with *C*. *dichromophloia* in the current APC.

^v^ Collected as *Corymbia semiclara* K.D.Hill & L.A.S.Johnson, a name that is synonymised with *C*. *hamersleyana* in the current APC.

^vi^ Collected as *Corymbia trachyphloia* subsp. *amphistomatica* K.D.Hill & L.A.S.Johnson, a name that is synonymised with *C*. *trachyphloia* in the current APC.

## Materials and methods

### Taxon sampling for chloroplast DNA study

Samples and sequences of chloroplast DNA used in this study are listed in Table 1. Names of species and infraspecific taxa generally follow the Australian Plant Census [1], and authorities are only given in the text for species names not listed in Table 1. Taxonomic works used to identify samples were [2,8,18]. For *Corymbia*, our sampling included 55 of the 97 species accepted by the Council of Heads of Australasian Herbaria (CHAH) [1], including all of the subgenera and sections recognised in the classification of [6], and with 19 species represented by at least two accessions. Sampling for *Angophora* included eight of the ten species recognised by CHAH [1], four of which were represented by at least two accessions. We also included sequences for 31 species of *Eucalyptus* and outgroups *Allosyncarpia* and *Stockwellia* from [41]. In total, the analysis included 123 accessions, of which 84 were newly sequenced for this study.

### DNA isolation from silica dried leaves

For the cpDNA study, total genomic DNA (gDNA) was extracted from ca. 80 mg of recently collected leaf tissue (no older than one year) using a modified CTAB DNA extraction protocol [44,45]. Older silica-dried collections were difficult to extract suitable DNA from, probably due to chemical DNA degradation in this plant group rich in secondary metabolites. The CTAB lysing buffer (2% w/v cetyltrimethylammonium bromide (CTAB), 2% w/v polyvinylpyrrolidone 40,000 (PVP–40), 1.4 M NaCl, 20 mM EDTA, 100 mM Tris–HCl pH 8.0) was modified by addition of 0.6% v/v each of 2–mercaptoethanol, RNase A, and proteinase K per sample. Further modifications to the CTAB extraction protocol included a sucrose/Tris/EDTA (STE) wash (8% w/v sucrose, 1 M Tris–HCl pH 7.0, 0.5 M EDTA) before lysis using 1 mL of STE per 80 mg of ground plant tissue [46]. The STE solution was discarded after centrifugation at 5,000 rpm for 10 min, and the pellet suspended in 700 μL of preheated (65°C) CTAB lysate buffer. After adding 110 μL bovine serum albumin (BSA)/NaCl (1:10, 4% BSA:5 M NaCl) to each sample, they were left to incubate for ca. 16 hrs at 60°C. Two 2/3 volume chloroform extractions were done, centrifuging for 10 min at 14,800 rpm for the first and then 8 min at the same speed for the second. DNA was precipitated with 2/3 volume of 100% isopropanol (room temperature). After 30–60 min incubation at room temperature, the DNA was centrifuged into a pellet at 14,800 rpm for 15 min and washed twice with 70% ethanol after discarding the isopropanol. DNA was resuspended in 100 μL TE pH 8.0 (10 mM Tris–HCl:1 mM EDTA pH 8.0) after leaving the pellet to dry overnight to allow all of the ethanol to evaporate. DNA quantity and quality were checked with Nanodrop 2000 (NanoDrop Products) and Qubit 2.0 fluorometer (Invitrogen) instruments and visualised by electrophoresis (1.5% agarose gel) with ethidium bromide.

### DNA library construction and sequencing

This section details a relatively cost-effective library preparation protocol at ca. AUD 35 per sample using no proprietary kits. All reagents are from New England BioLabs (NEB) if not stated otherwise. Immediately before sonication, a DNA aliquot was washed with ethanol/sodium acetate (5.5:1, 100% ethanol:2.4M NaAc) at a 1:4.7 DNA:wash solution volume, and then centrifuged at 14,800 rpm for 10 min. After discarding the wash solution, the resulting pellet was washed with 70% ethanol and resuspended in 100 μL 1 M Tris–HCl pH 8.0. DNA was quantified with a Qubit 2.0 (Invitrogen), and an aliquot of 3 μg of gDNA per sample was brought to 115 μL with ultrapure H_2_O. The DNA was sonicated for 50 sec with a S220 Focused-ultrasonicator (Covaris) set to 6–8°C, 120W peak incident power, 200 cycles per burst, and on duty cycle 5%, aiming for 800 bp mean fragment size.

The sonicated samples (100 μL each) were cleaned using Serapure SPRI beads [47] at a 0.6:1.0 beads:sample ratio to remove short fragments (<300 bp) by incubating this mixture for 20 min at room temperature, immobilising beads on a 96S super magnet plate (Alpaqua) for 15 min, discarding the supernatant and washing with 170 μL 80% ethanol, and then leaving the magnet-trapped beads to air dry for 2 min.

NEBNext End Repair Module produced blunt ends on the fragmented DNA by eluting the DNA from the magnet-trapped beads and incubating with 2.0 μL 10 × reaction buffer, 0.4 μL enzyme mix, and 17.6 μL ultrapure H_2_O per sample at 20°C for 60 min. Samples were again purified using the Serapure SPRI beads by adding 50 μL PEG:NaCl (20% PEG w/v:5 M NaCl) and 50 μL 100% isopropanol to each sample, incubating this for 15 min at room temperature, and washed using 80% ethanol as in the above steps. Then dA tails were attached to the fragments using 0.5 μL 10 mM deoxyadenosine 5'-triphosphate, 0.4 μL Klenow Fragment, 2.0 μL NEB 10 × Buffer 2, and 17.1 μL ultrapure H_2_O per sample to elute the DNA and incubated at 37°C for 60 min followed by 65°C for 20 min. Per sample, 2 μL of 25 μM multiplex hairpin adaptors (top plus bottom strands) in 10 mM Tris–HCl pH 8.0 [47] were ligated to the DNA fragments with 0.25 μL10 mM ATP, 0.4 μL T4 DNA ligase, 0.8 μL T4 DNA ligase buffer, and 6.55 μL ultrapure H_2_O at 12°C overnight and then 10 min of 65°C to stop the reaction. Exonuclease digestion degraded all non-competent molecules with 0.5 μL 10 × NEB Buffer I, 0.25 μL Lambda Exonuclease, 0.25 μL Exonuclease I, and 4.0 μL ultrapure H_2_O per sample at 37°C for 2 hrs, and 80°C for 20 min. After the digestion, samples were cleaned on the magnetic plate as before, but using 80 μL 20% PEG:5M NaCl and 80 μL 100% isoproplanol per sample. Samples were eluted with 60 μL 10 mM Tris–HCl pH 8.0 and stored at 4°C until q-PCR titration, after which the libraries were kept at -20°C for long term storage.

For titration, a 20 μL q-PCR reaction, using 5 μL of each 800 bp library as template, 0.5 μL 10× SYBR Green (Thermo Fisher Scientific), 10.0 μL Kapa HiFi HotStart ReadyMix (KAPA Biosystems), 2.5 μL ultrapure H_2_O, and 1.0 μL each of 5 μM TRUESEQ (Illumina) compatible PE primers [47] was run to 20 cycles on a BioRad CFX q-PCR machine. Settings for the q-PCR were 30 sec of 98°C for denaturation and 20 cycles of 98°C for 10 sec, 67°C for 30 sec, and 72°C for 30 sec. Once the sample appropriate cycle number was determined from this q-PCR, a 40 μL reaction and including sample-specific PE primers including indexing barcodes to allow pooling of multiple samples per sequencing was run using the same settings as before.

Samples were pooled and quality checked either with an Agilent Bioanalyser DNA1000 chip system (Agilent) for HiSeq 1500 (Illumina) sequencing or a 2200 Tape Station using the D1000 kit (Agilent) and Qubit 3.0 (Invitrogen) for sequencing on a NextSeq 500 machine (Illumina). A 250 cycle (2 × 125 paired end reads) kit (Illumina) was used for the former or a 300 cycle (2 × 150 paired end reads) kit (Illumina) for the latter sequencer.

### Sequence trimming, quality control, read mapping and chloroplast sequence assembly

Base calling and quality filtering was done with Illumina pipeline software (v.1.7 or later) and samples were pre-processed with custom scripts at the Walter and Eliza Hall Institute of Medical Research (WEHI) sequencing facility.

The new sequences were assessed, trimmed and assembled with CLC Genomics Workbench v. 9.5.1 and 9.5.2 (Qiagen) and the CLC Workflow is available as supplementary material (S1 File). Paired-end reads were paired and reads shorter than 15 or longer than 1000 bp were discarded. Reads below PHRED score 20 were also discarded. The fraction of low quality bases that were allowed in a read was 5%.

The quality-filtered and paired reads were mapped against a reference chloroplast genome (*Eucalyptus globulus*, GenBank accession: NC_008115.1). Sequence coverage was generally sufficient to unambiguously assemble most of the chloroplast genome for each sample, but all genomes included some regions with low coverage of mapped reads. A consensus sequence of the mapped assembly was created by removing regions with low coverage and inserting ambiguity codes for bases with more than one possible nucleotide using a threshold of 50% and ‘maximum number of ambiguous nucleotides allowed after trimming = 2’.

### Sequence alignment and phylogenetic analyses of chloroplast DNA

All newly generated chloroplast sequences included in the study were aligned with MAFFT v.7.299b [48] and the fast and progressive method FFT-NS-2, suitable for large alignments. One inverted repeat region (IRa) was excluded from the alignment. The alignment was viewed with SeaView v.4.6 [49] or Mesquite v.3.10 [50] and subsequently processed with GBLOCKS v.0.91b [51] using default parameters, which stringently trims alignments allowing no gaps. Hence, the final dataset only included regions with sequence coverage for all samples, and all indels were removed.

We used jModelTest v.2.1.10 [52,53] and the AIC and BIC criteria, to estimate the model of nucleotide substitution that best fits the chloroplast data. The maximum likelihood analysis was done with Standard RAxML v.8.2.8 [54] with 1000 rapid bootstrap inferences and a thorough ML search under the GAMMA model of rate heterogeneity. The maximum parsimony analysis was done with PAUP* v.4.0a151 [55] with the following settings: all characters were treated as unordered and of equal weight. Heuristic searches employed tree-bisection-reconnection branch swapping and 1000 replicates of random stepwise additions. The number of bootstrap replicates was 1000 with one tree held at each step. Trees were viewed and exported for rendering in FigTree v.1.4.3 [56].

### Analysis of nuclear ribosomal DNA

For comparison with the cpDNA phylogeny, we combined sequences of the ITS regions of nuclear ribosomal DNA (nrDNA) from previous studies [6,26,29,30,31,57,58] for phylogenetic analyses. Separate analyses of these nrDNA sequences have not been presented in previous studies, with most including only a small number of *Corymbia* samples or, in the case of the largest study to date [31], also combining a subset of these sequences with cpDNA markers in analyses of a concatenated dataset. Our dataset included 66 accessions of *Corymbia* (representing all taxonomic sections), 15 of *Angophora* (9 of 10 species), 31 of *Eucalyptus* (the same species as in the cpDNA dataset, representing major lineages), two of *Stockwellia*, one of *Eucalyptopsis*, three of *Allosyncarpia*, and one of *Arillastrum* (used as outgroup). Partial sequences, or those identified as spacers associated with pseudogenes using established criteria [59,60,61,62], were excluded from analyses. We used existing nrDNA sequences for analyses, rather than assembling novel sequences from our current genomic data for *Corymbia*, because of the presence of substantial within-genome variation in our samples (in line with previous reports [38,59,60]), and associated difficulties in separating and assembling sequences of the various paralogues/alleles, which is a challenging task worthy of separate investigation and discussion. The nrITS sequences from GenBank were aligned using Geneious v.9.1.7 [63]. Model testing, ML and MP analyses were conducted as outlined above with the addition that gaps present in the nrDNA alignment were treated as missing data in the MP analysis.

## Results

### Analysis of chloroplast DNA

**Fig 3:**
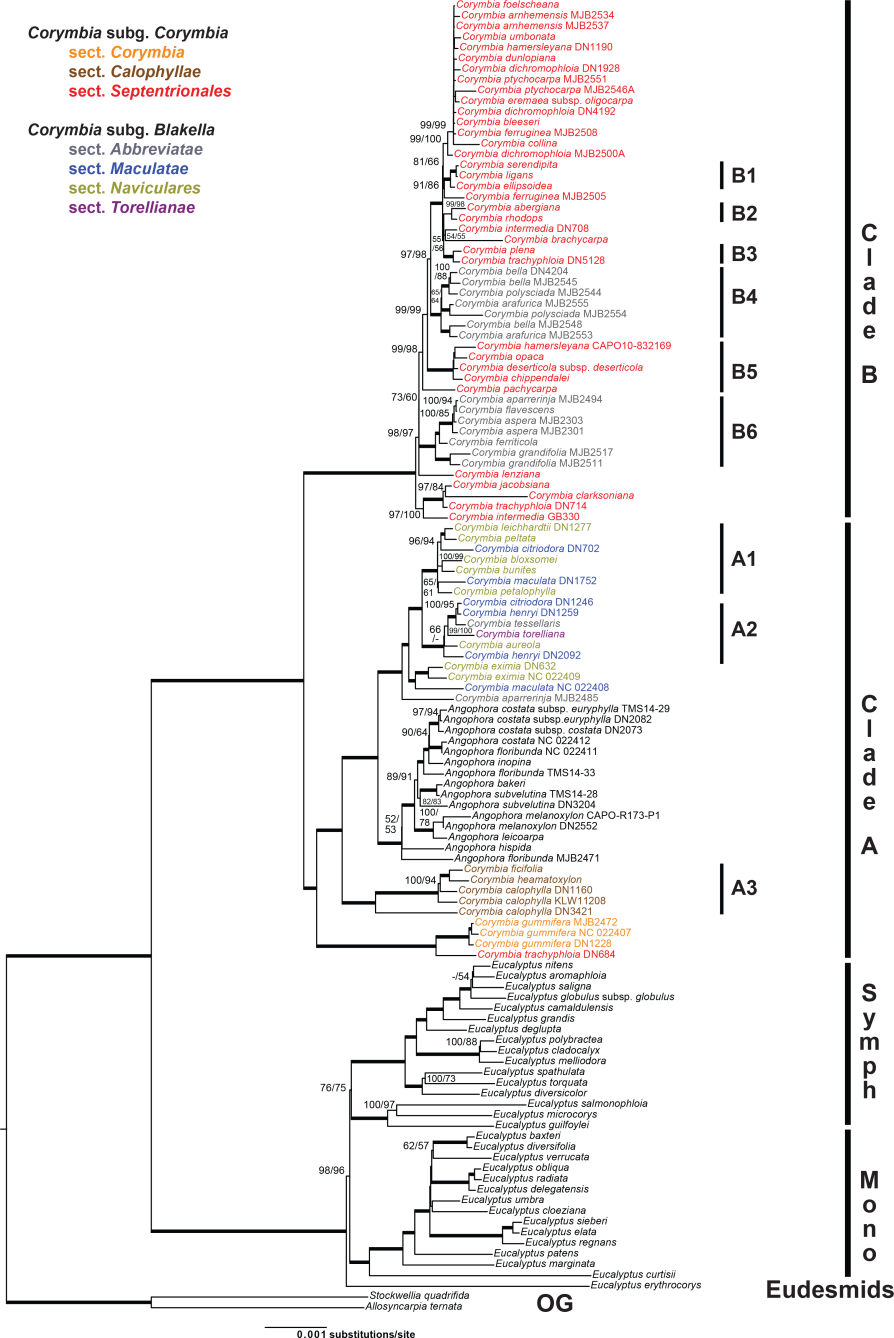
Phylogeny resulting from cpDNA analyses. Best-scoring maximum likelihood tree from a RAxML analysis (final ML optimization likelihood of -254343.104231) of a cpDNA dataset (121,016 base pairs, 10,847 distinct alignment patterns, 123 accessions) of eucalypts. Names of *Corymbia* species are colour-coded by taxonomic section as indicated. Labelling to the right of the tree indicates the outgroup (‘OG’), major groups of *Eucalyptus*, including subg. *Eudesmia* (‘Eudesmids’), the symphyomyrt clade (‘Symph’; including subgenera *Alveolata*, *Cruciformes Minutifructus* and *Syphyomyrtus*), and the monocalypt clade (‘Mono’; including subgenera *Acerosae*, *Eucalyptus* and *Idiogenes*), as well as subclades of *Corymbia* clustering by geographic proximity (A1–A3 and B1–B6) referred to in the text and Fig 4 (for clade A) and Fig 5 (for clade B). Species names of species represented by multiple accessions are followed by collection number for newly generated sequences or GenBank accession number (NC) for data generated for previous studies. Bootstrap support values are shown as percent maximum likelihood/maximum parsimony (MP mapped onto ML tree) with weighted edges indicating 100% support for both ML and MP. Support values <50% are omitted or dashed when the alternate analysis method had ≥50% support.

GBLOCKS eliminated 37% of the MAFFT alignment, resulting in 121,016 characters and 10,847 distinct alignment patterns in the final alignment (see supplementary material S2 File). Both, AIC and BIC from jModelTest indicated the General Time Reversible model using gamma and invariant sites (GTR+I+G) as the best fit. Final ML Optimization Likelihood was -254343.104231. The maximum parsimony analysis had 3771 parsimony informative characters, 4116 variable but uninformative characters, and resulted in 12 trees with length = 10413, consistency index = 0.81, retention index = 0.96. Topologies of the ML and MP trees were similar (Fig 3), and most nodes had 85–100% bootstrap support (BS) for both ML and MP analyses, with the backbone, in particular, well supported.

Rooting the trees with *Allosyncarpia* and *Stockwellia* recovered relationships of a monophyletic *Eucalyptus* as sister to *Angophora* + *Corymbia*. *Eucalyptus* is composed of three clades corresponding to ‘Eudesmids’ (subg. *Eudesmia*, represented by *E*. *erythrocorys*) subtending a well-supported clade of ‘Monocalypts’ *sensu* [41] (including representatives of subg. *Acerosae* [*E*. *curtisii*], subg. *Idiogenes* [*E*. *cloeziana*], and subg. *Eucalyptus* [remainder of that clade]), which were sister to a ‘Symphyomyrt’ clade (including members of subg. *Alveolata* [*E*. *microcorys*], subg. *Cruciformes* [*E*. *guilfoylei*], and subg. *Minutifrucuts* [*E*. *deglupta*] nested in subg. *Symphyomyrtus* [remainder of that clade]) that was moderately supported with BS ML/MP 76/75%.

*Corymbia* is paraphyletic because of the inclusion of *Angophora* in one *Corymbia* clade (clade A; Fig 3). Furthermore, subgenera *Blakella* and *Corymbia*, most non-monotypic sections (*Abbreviatae*, *Maculatae*, *Naviculares*, *Septentrionales*), and several species including more than one accession here are not monophyletic. In addition to *Angophora*, clade A includes a basal grade of a few species of red bloodwoods from southern Australia that do not group with all other red bloodwoods in clade B. The red bloodwoods in clade A include *C*. *gummifera* (monotypic sect. *Corymbia*) from south-eastern Australia and *C*. *calophylla*, *C*. *ficifolia*, and *C*. *haematoxylon* that correspond to sect. *Calophyllae* from south-western Western Australia (Fig 1). Clade A also includes the yellow bloodwoods (sect. *Naviculares*), spotted gums (sect. *Maculatae*), cadagi or *C*. *torelliana* (monotypic sect. *Torellianae*), and two ghost gums (sect. *Abbreviatae*). Although the latter all form a clade, spotted gum and yellow bloodwood species are interdigitated and taxonomic sections based on morphology do not form groups here. The ghost gums *sensu* Parra-Osorio et al. [6] are polyphyletic, because clade B also includes two separate clades of sect. *Abbreviatae*. In addition, clade B contains most of the red bloodwood species (sect. *Septentrionales*), in which the ghost gums are embedded.

Of 23 species of *Angophora* and *Corymbia* represented in the dataset by two or more samples, only four species (*A*. *melanoxylon*, *C*. *eximia*, *C*. *grandifolia*, and *C*. *gummifera*) are resolved as monophyletic, whereas most are indicated as paraphyletic or polyphyletic, and two species (*C*. *trachyphloia* and *C*. *aparrerinja*) have accessions split between the two major ingroup clades (clades A and B). This widespread incongruence between morphological and chloroplast data likely points to a complex evolutionary history in this group. In conclusion, both hypotheses to be tested, 1) that *Corymbia* is monophyletic and 2) that the currently recognised subgenera of *Corymbia* are monophyletic, are not supported based on the chloroplast data.

**Fig 4:**
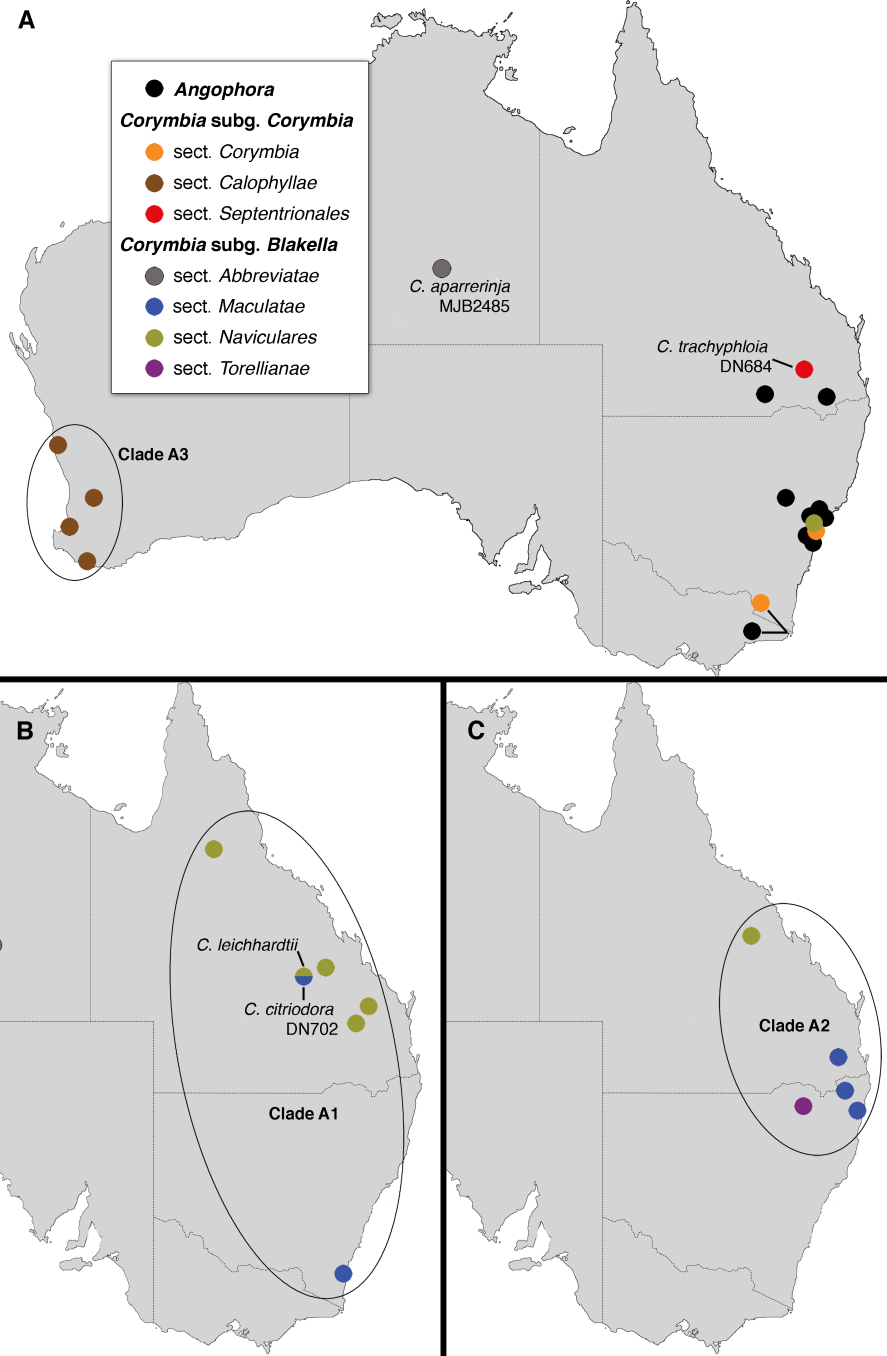
Distribution of samples placed in clade A in the cpDNA phylogeny (Fig 3). Colour coding of groups matches that used in other figures. Accession details are shown for four samples mentioned in the text.

**Fig 5:**
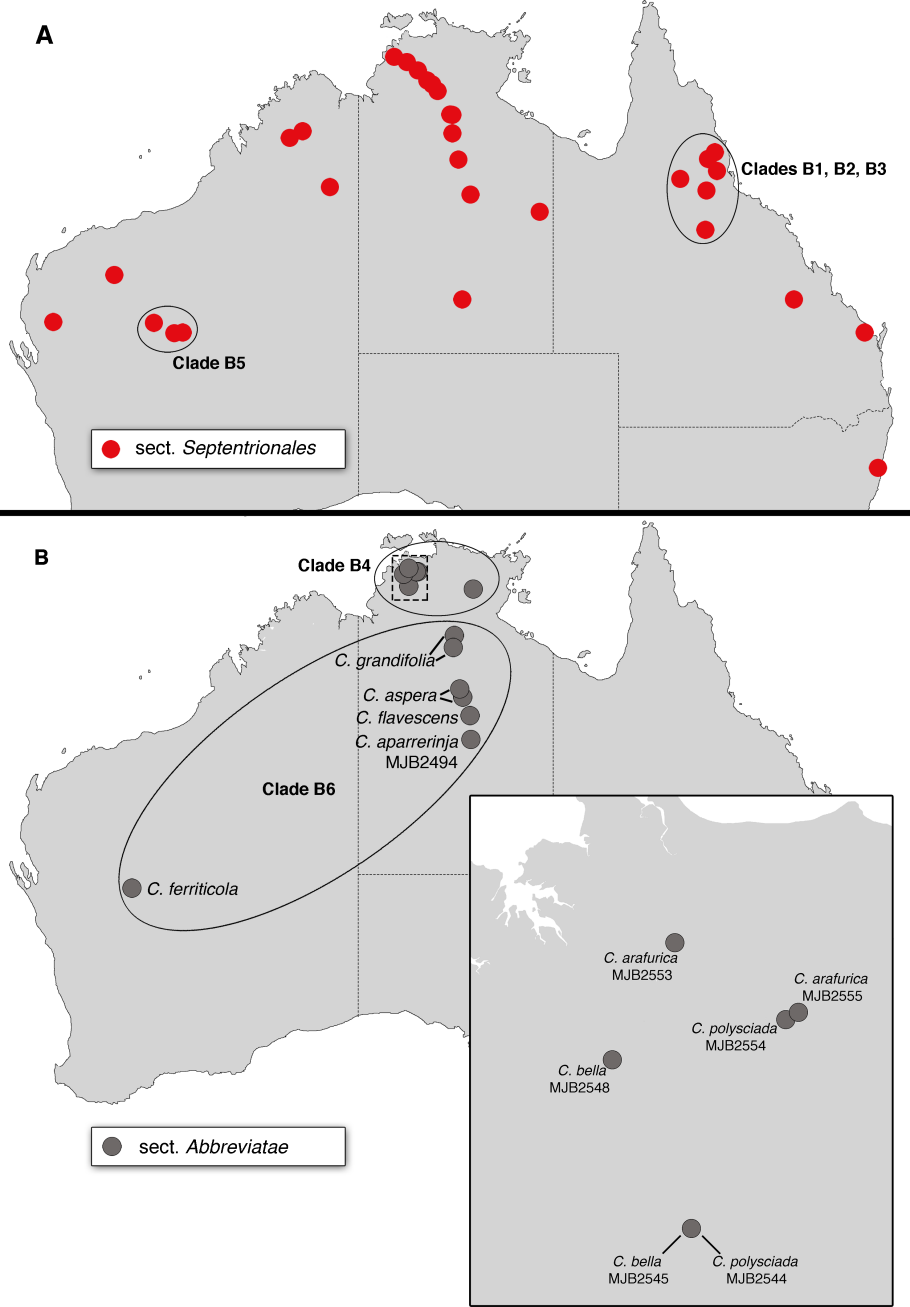
Distribution of samples placed in clade B in the cpDNA phylogeny (Fig 3). (A) Samples classified in sect. *Septentrionales*; (B) samples classified in sect. *Abbreviatae*, with inset in lower right showing detail for area outlined by dashed rectangle. Details are shown for some clades, species, and accessions mentioned in the text. Colour coding of groups matches that used in other figures.

Some phylogenetic signal in the cpDNA data is geographic, and Fig 4 (for clade A) and Fig 5 (for clade B) illustrate the proximity of accession localities that form subclades within the major two ingroup clades. For example, Fig 4B shows the geographic proximity of accessions included in clade A1 (Fig 3), which is composed of members of sections *Maculatae* and *Naviculares*, and Fig 5 shows geographic groups within clade B that each include a mix of species from different taxonomic series (see Table 1) within sections *Septentrionales* and *Abbreviatae*.

### Analysis of nuclear ribosomal DNA

**Fig 6:**
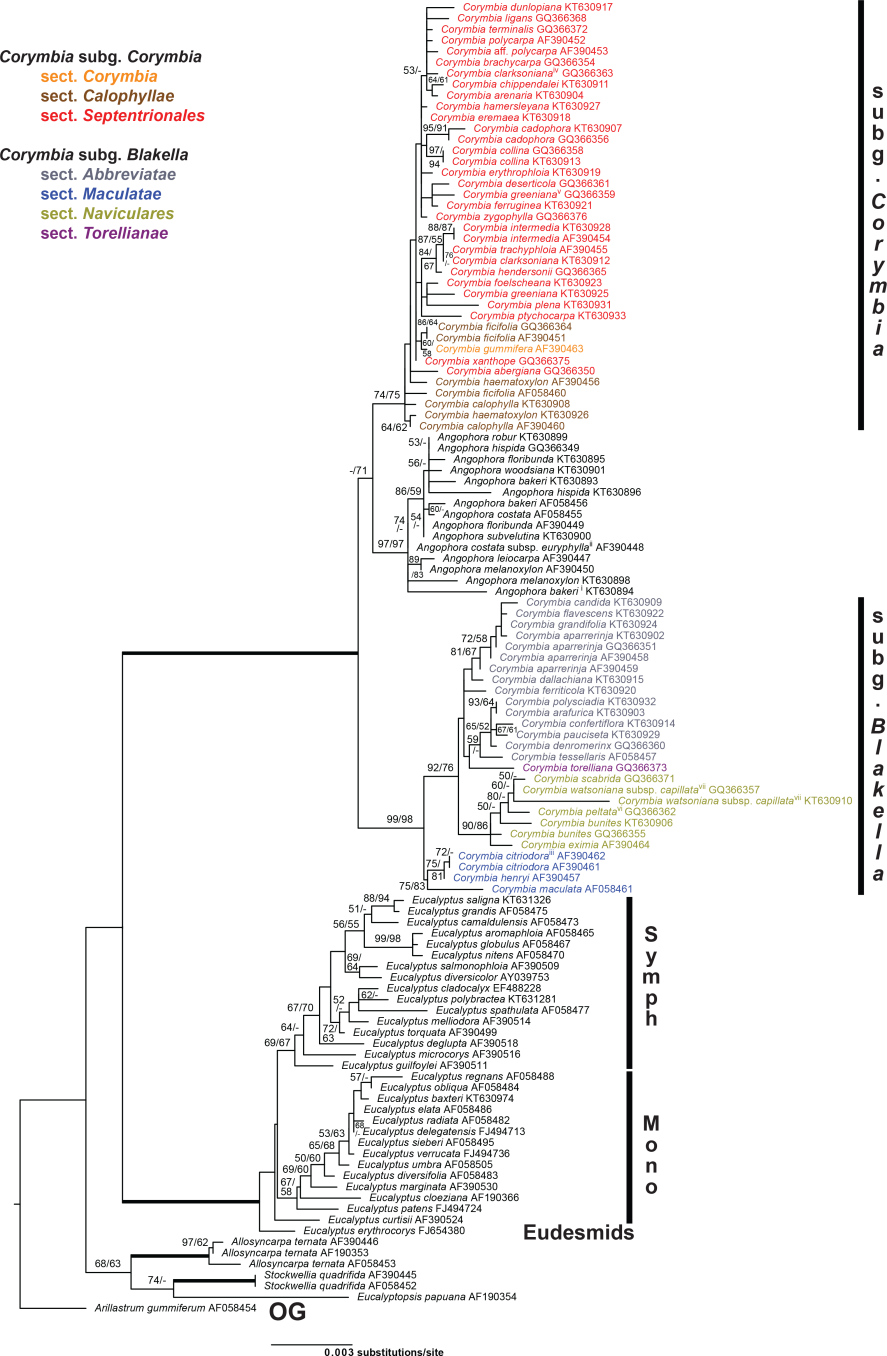
Phylogeny resulting from nrDNA analyses. Best-scoring maximum likelihood tree from a RAxML analysis (final ML optimization likelihood of -4709.258464) of a nrITS dataset (663 base pairs, 337 distinct alignment patterns, 119 accessions) of eucalypts. The outgroup (OG), major groups in *Eucalyptus* (‘Eudesmids’, ‘Monocalypts’ [Mono], and ‘Symphyomyrts’ [Symph]), subgenera and infrageneric groups of *Corymbia* are indicated with bars or colouring scheme (see textbox) on the tree. Species names are followed by GenBank accession numbers. Bootstrap support values are shown as percent maximum likelihood/maximum parsimony (MP mapped onto ML tree) with weighted edges indicating 100% support for both ML and MP. Support values <50% are omitted or dashed when the alternate analysis method had ≥50% support. ^i^ Identified in GenBank (GB) as *Angophora exul* K.D.Hill, a name that is synonymised with *A*. *bakeri* in the current Australian Plant Census (APC) [1]. ^ii^ Identified in GB as *Angophora euryphylla* (G.J.Leach) L.A.S.Johnson & K.D.Hill, a name that is synonymised with *A*. *costata* subsp. *euryphylla* in the current APC. ^iii^ Identified in GB as *Corymbia variegata* (F.Muell.) K.D.Hill & L.A.S.Johnson, a name that is synonymised with *C*. *citriodora* in the current APC. ^iv^ Identified in GB as *Corymbia dolichocarpa* (D.J.Carr & S.G.M.Carr) K.D.Hill & L.A.S.Johnson, a name that is synonymised with *C*. *clarksoniana* in the current APC. ^v^ Identified in GB as *Corymbia dampieri* (D.J.Carr & S.G.M.Carr) K.D.Hill & L.A.S.Johnson, a name that is synonymised with *C*. *greeniana* in the current APC. ^vi^ Identified in GB as *Corymbia dimorpha* (Brooker & A.R.Bean) K.D.Hill & L.A.S.Johnson, a name that is synonymised with *C*. *peltata* in the current APC. ^vii^ Identified in GB as *Corymbia catenaria* K.D.Hill & L.A.S.Johnson, a name that is synonymised with *C*. *watsoniana* subsp. *capillata* in the current APC.

The nrDNA dataset included 663 aligned bases and 337 distinct alignment patterns (see supplementary material S3 File). AIC from jModelTest indicated a General Time Reversible model using gamma distribution of rates and a proportion of invariant sites (GTR+I+G), used for analysis here, and BIC indicated GTR+G as the best fit. Final ML optimization likelihood was -4709.258464. The MP analysis included 170 parsimony informative characters and the alignment had 90 variable but uninformative characters. Maximum parsimony analysis resulted in 1610 trees with length = 644, consistency index = 0.55, and retention index = 0.90. Topologies of the ML and MP trees were similar, and the ML tree is shown here, with MP bootstrap support values mapped onto it (Fig 6).

Analyses strongly supported the monophyly of *Eucalyptus* and of *Corymbia* + *Angophora* (both with ML/MP BS of 100%). Relationships within *Eucalyptus* were similar to those in the cpDNA tree, in that they resolved the main ‘Monocalypt’ and ‘Symphyomyrt’ clades, subtended by subg. *Eudesmia* (*E*. *erythrocorys*), although the position of monotypic subg. *Acerosae* (*E*. *curtisii*) was not resolved with support.

*Corymbia* was resolved as paraphyletic with respect to *Angophora*, and that relationship received weak to moderate support (BS of<50% for ML and 71% for MP). Within *Corymbia*, there was support for the monophyly of subg. *Corymbia* (ML/MP BS of 74/75%) and subg. *Blakella* (BS 99/98%). Monophyly was also supported for *Corymbia* sections *Maculatae* (BS 75/83%) and *Naviculares* (BS 90/86%). Relationships of other *Corymbia* sections represented by more than one species were generally poorly supported, i.e.: sect. *Septentrionales* was resolved as paraphyletic, but with < 50% ML or MP BS; sect. *Calophyllae* was resolved as paraphyletic, on account of placement of *C*. *gummifera* (monotypic sect. *Corymbia*) with two samples of *C*. *ficifolia* with weak support (BS 60/58%); sect. *Abbreviatae* was resolved as paraphyletic with respect to monotypic sect. *Torellianae*, but only in the ML tree and with <50% bootstrap support. In conclusion, support was mixed for the hypotheses being tested here, 1) that *Corymbia* is monophyletic and 2) that the currently recognised subgenera of *Corymbia* are monophyletic. For hypothesis 1), the data are largely equivocal, there being only weak support for the nesting of *Angophora* in *Corymbia* (<50% BS for ML and 71% for MP); for hypothesis 2), the current subgeneric classification of *Corymbia* was moderately to strongly supported.

## Discussion

The relationships within *Eucalyptus* generally confirm those of previous HTS studies [41] and therefore, our results for relationships among *Eucalyptus* will not be discussed further as our focus here is on *Corymbia* and *Angophora*.

### Why are cpDNA relationships incongruent with nrDNA relationships and infrageneric classification of *Corymbia*?

A key result of the current study is that cpDNA relationships in *Corymbia* (Fig 3) are largely incongruent with the current circumscriptions of subgenera and sections, and with relationships inferred based on nrDNA (Fig 6). Such incongruence could occur if: A) current infrageneric groups are poorly defined and in need of taxonomic revision; B) the nrDNA gene tree does not accurately reflect phylogenetic relationships, e.g., as a result of mixing of orthologous and paralogous copies of this multi-copy cistron [60,64,65]; C) the cpDNA gene tree does not accurately reflect the phylogenetic relationships of taxa, e.g., as a result of processes such as incomplete lineage sorting [66,67,68] or chloroplast capture resulting from hybridisation and introgression [69,70]. We infer that the observed incongruence is consistent with the last explanation and, in particular, points to historical hybridisation and cpDNA introgression between lineages, as outlined below.

In conflict with the cpDNA gene tree, evidence for the monophyly of major infrageneric groups in *Corymbia* (subgenera and sections) comes from a general concordance between phylogenies based on nrDNA sequences (e.g., [6,29,30]) and morphologically defined infrageneric groups. For instance, the analysis of nrDNA presented here (Fig 6), based on ITS sequences, supports monophyly of the two subgenera (subg. *Corymbia* and subg. *Blakella*), the yellow bloodwoods (sect. *Naviculares*) and the spotted gums (sect. *Maculatae*), and it does not strongly contradict the monophyly of the ghost gums (sect. *Abbreviatae*). Most of these groups have historically been recognised on morphological grounds, although at varying taxonomic levels (e.g., [2,3,4]). Such concordance, from independent data sources, provides support for the notion that, on the whole, these taxa represent phylogenetic groups. Thus, it is striking that, among the molecular phylogenetic studies of *Corymbia*, it is only those including chloroplast data that show strongly supported nodes in conflict with the recognition of these groups (current study and [31,40]). Understanding the reasons for this conflict is central to gaining insight into the evolutionary history of *Corymbia*, and to properly testing its classification.

Chloroplast capture and incomplete lineage sorting are two processes commonly inferred to account for incongruence in plants between chloroplast DNA relationships and nuclear DNA phylogenies/morphological taxonomy. The relative importance of these processes can be difficult to infer or disentangle [71], but some clues can come from knowledge of the reproductive biology of the plants and of geographic patterns of DNA sequence variation. In terms of reproductive biology, a capacity to hybridise and interbreed is a necessary pre-requisite for the transfer of chloroplasts between lineages. In terms of geography, introgression necessarily occurs at particular locations, and can lead to geographic clustering in the sharing of related chloroplast sequences between species [71]. In contrast, such geographic clustering might not be expected in cases where incongruence with taxonomy results from incomplete lineage sorting of chloroplast genomes (e.g. [72]).

**Fig 7:**
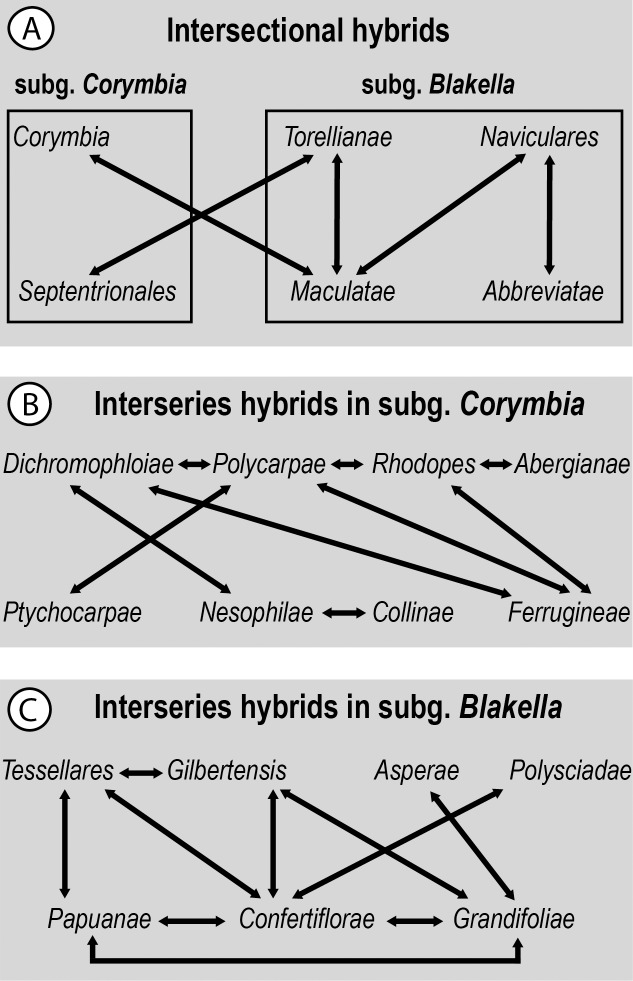
Summary of hybridisation between infrageneric groups of *Corymbia* inferred by previous studies [2,73,77]. Arrows connect taxa with inferred hybrids. (A) Inferred intersectional hybrids; (B) interseries hybrids in subg. *Corymbia*; (C) interseries hybrids in subg. *Blakella*.

Although incomplete lineage sorting cannot be excluded as an explanation for aspects of cpDNA relationships in *Corymbia*, it seems likely from the reproductive biology of these trees (and that of other eucalypt genera), together with geographic patterns of cpDNA variation, that the observed patterns are largely consistent with a history of hybridisation and introgression. In terms of reproductive compatibility, pre-zygotic barriers to reproduction have been reported between some *Corymbia* species [73], but all members of *Corymbia* investigated so far have the same chromosome number (2*n* = 22; [74,75]). Both morphological variation and experimental crosses [2,73,76,77] provide evidence of substantial potential for hybridisation between species classified in different series, sections, and subgenera (summarised in Fig 7). Given this capacity for hybridisation across infrageneric groups, it seems likely that the taxonomic incongruence of cpDNA relationships in *Corymbia* could reflect similar processes to those seen in the better studied *Eucalyptus*. In *Eucalyptus*, such incongruence is clearly evident, with chloroplast variation commonly reflecting geography, rather than taxonomy, and widespread regional introgression of cpDNA between species, series, and sections is regularly inferred [71,78,79,80,81,82,83,84,85].

Geographic patterns in *Corymbia* observed here (Figs 4 and 5) provide support for a history of cpDNA introgression between species, in the form of geographically clustered cpDNA clades shared among taxa that would be considered distinct lineages based on morphological or nrDNA evidence. Such patterns are consistent with cpDNA introgression between species across a range of morphological infrageneric groups.

Some of the clearest geographic patterns in our dataset relate to geographic cpDNA clades shared among species classified, on the basis of morphology [2], in different taxonomic series (Table 1) within the same section. For example, within sect. *Septentrionales* three geographic clades in north Queensland each contain a mixture of species placed in different taxonomic series, i.e., clade B1 (containing members of ser. *Arenariae*, *Polycarpae*, and *Dichromophloiae*; Fig 3), clade B2 (containing members of ser. *Rhodopes* and *Abergianae*), and clade B3 (containing members of ser. *Trachyphloiae* and *Polycarpae*). Likewise, clade B5 (including members of ser. *Dichromophloiae* and ser. *Ferrugineae*) is geographically clustered in the Mid-West region of Western Australia. Apart from the monotypic ser. *Abergianae*, series with species falling in clades B1, B2, B3, and B5 are also distributed across other clades in the phylogeny and hence not monophyletic (e.g., *C*. *dichromophloia*, *C*. *hamersleyana*, and *C*. *trachyphloia*). Similar patterns are also seen in clades of sect. *Abbreviatae*, where clade B4 includes intermixed representatives of series *Polysciadae* and *Papuanae*, and clade B6 includes intermixed members of ser. *Grandifoliae* and *Asperae*. Interestingly, in clade B4, the morphologically distinctive species *C*. *polysciada* (ser. *Polysciadae*) is strongly supported as polyphyletic, with the two accessions each having cpDNA haplotypes more closely related to those of closely occurring species from ser. *Papuanae* (compare Fig 3 and inset on Fig 5B). Although each of these morphologically defined series present in clades B1–B6 cannot be assumed *a priori* to represent monophyletic groups (e.g., there is no nuclear genetic evidence to support their monophyly), the presence of geographically distinct chloroplast clades found across morphologically distinctive taxa suggests a history of local chloroplast introgression between lineages, much as seen in *Eucalyptus*.

Geographic patterns in cpDNA clades shared across different taxonomic sections or subgenera are less clear than those among series, but geographic links can still be discerned. For instance, clade A1 (Fig 3) although ranging from north Queensland to southern New South Wales in eastern Australia, has a cluster of samples from south-east Queensland including one of *C*. *citriodora* (sect. *Maculatae*) that groups in the cpDNA gene tree as sister to a clade including a sample of *C*. *leichhardtii* (sect. *Naviculares*) that occurs nearby (Fig 4B). Similarly, clade A2, ranging from northern New South Wales to mid-east Queensland (Fig 4C), includes, in reasonably close geographic proximity, members of sect. *Maculatae*, together with *C*. *torelliana* of monotypic sect. *Torellianae*. However, the representative of sect. *Naviculares* in this clade is geographically more distant, and the representative of sect. *Abbreviate* could not be mapped because the sample is of unknown provenance. At least the geographic and cpDNA association of *C*. *torelliana* and the three samples of sect. *Maculatae* is consistent with known capacity for these groups to interbreed [73,76]. Again, these groups (although not these species) have previously been reported to hybridise ([77]; Fig 7).

A striking feature of the cpDNA tree is placement of two clades of ghost gums, subg. *Blakella* sect. *Abbreviatae* (clades B4 and B6; Fig 3), within the large clade B that otherwise includes almost all samples of the red bloodwood group subg. *Corymbia* sect. *Septentrionales*. This contrasts with phylogenetic analyses of nrDNA sequences (Fig 6 and [6,25,27,29,30]), in which sect. *Abbreviatae* groups with other members of subg. *Blakella*. The geographic clustering of clades B4 and B6, and their distribution in areas where members of sect. *Septentrionales* are common, especially in the Northern Territory, is consistent with these clades reflecting historical chloroplast transfer from red bloodwoods to ghost gums, e.g., potentially two distinct events of chloroplast transfer, in each case with a red bloodwood as the initial maternal parent. Hybridisation between these groups has not, to our knowledge, been reported (e.g. Fig 7; [2,73,77]).

It is worth noting that taxon sampling for the current study was designed primarily to sample across the major taxonomic groups of *Corymbia*, to assess their relationships on the basis of chloroplast sequences, and it was not designed specifically to assess geographic patterns of chloroplast variation. As such, there are substantial geographic distances between many samples, and the spread of samples is quite unbalanced, e.g., with only one sample of sect. *Abbreviatae* from eastern Australia (*C*. *tessellaris*, of unknown wild provenance), and none from the north-west of Western Australia. The inferences here of historical chloroplast introgression between major taxonomic groups, especially subgenera and some sections, are consistent with the observed patterns of cpDNA variation, and knowledge of bloodwood reproductive biology, but remain speculative. Our study provides insight into the potential importance of this process in the evolutionary history of *Corymbia*, but more detailed studies using fine-scale geographic sampling, including multiple replicates of species, are necessary both to properly test for the presence of chloroplast introgression, and to more fully appreciate its significance and any accompanying patterns of nuclear gene flow.

### Chloroplast DNA relationships in *Angophora*

The genus *Angophora* was strongly supported as monophyletic, as universally found in all molecular phylogenetic studies of eucalypts that have sampled two or more *Angophora* species (e.g. [6,27,29,30,31]). Most previous studies have included only a small number of exemplars from *Angophora* and thus both species limits, which differ between some treatments [1,18,86,87], and the proposed infrageneric classification [87], have not been critically tested by molecular data. Our cpDNA study included multiple accessions of four species, three of which were strongly indicated as paraphyletic or polyphyletic (Fig 3); the one species that was resolved as monophyletic, *A*. *melanoxylon*, was represented by only two samples from the same geographic area, near St George in south-east Queensland. The sample set here is small (15 samples from 8 species), and was not collected to assess geographic variation within/between taxa, but it seems reasonable that taxonomic incongruence with cpDNA variation in *Angophora*, as in *Eucalyptus* and *Corymbia* (see above) reflects, at least in part, a history of cpDNA introgression between species. Consistent with this is the resolution of *A*. *subvelutina* as paraphyletic (in particular, with one sample shown as sister to a closely co-occurring sample of *A*. *bakeri*), and the polyphyly of *A*. *floribunda*, in which the northern-most sample of known provenance (TMS14–33; Table 1) falls in a clade of other samples from nearby areas, and is well separated in the phylogeny from the southernmost sample (MJB 2471). An influence of chloroplast introgression on this gene tree would be consistent with the observation of Leach [86] that hybridisation between species of *Angophora* "… has been observed in virtually all combinations that are geographically or ecologically conceivable". As with *Corymbia*, fine-scale studies of cpDNA variation could be used to test for both the presence and extent of cpDNA introgression amongst *Angophora* species.

### Implications for genus-level taxonomy

A primary aim of this study was to use HTS chloroplast data, from a broad sample of infrageneric groups, to test the monophyly of the bloodwood genus *Corymbia* as currently circumscribed. The inferred chloroplast relationships strongly support nesting of *Angophora* in *Corymbia* thus making it paraphyletic (Fig 3), as also shown, with less support, in previous cpDNA studies using either more limited taxon sampling or more limited sampling of the chloroplast genome [26,28,31,40,41]. However, given the clear incongruence between the cpDNA gene tree and taxonomic boundaries that are otherwise supported by both morphological characters and analyses of nrDNA, and given the likely influence of historical cpDNA introgression between lineages (discussed above), the cpDNA data, on their own, do not provide a sound basis for assessing generic limits in this group. It is worth noting that it seems unlikely that the close cpDNA relationship of *Angophora* to some groups of *Corymbia* could be directly attributed to cpDNA introgression (at least not recently), because *Corymbia*–*Angophora* hybrids have not been reported (e.g. [2,73,77]), despite common co-occurrence of species [2] and attempts at artificial crosses (e.g. [73]).

To make sound taxonomic decisions, especially regarding the limits of genera and subgenera, among the bloodwoods and their relatives, better knowledge of relationships based on nuclear DNA sequences is essential. Analyses of nuclear DNA datasets have so far been limited to the ITS, ETS and 5S regions of nrDNA [6,25,27,29,30,38] and a small number of microsatellite markers [39], and have given mixed support for the monophyly/paraphyly of *Corymbia*. The nrDNA analyses here (Fig 6), for instance, using only ITS data, show 71% BS for the nesting of *Angophora* in *Corymbia* in the MP analysis but <50% support in the ML analysis, leaving open the possibility that *Angophora* might be sister to a monophyletic *Corymbia*. More thorough assessment of relationships will require analysis of more substantial datasets, for which there are now good prospects using HTS methods [37].

Even if *Angophora* proves to be nested in *Corymbia* based on nuclear data, placing them together in one genus might not be the best taxonomic solution for this group. Instead, raising one or more of the infrageneric groups of *Corymbia* to genus rank might be a better solution for recognising monophyletic, morphologically diagnosable (less heterogeneous) groups and minimising taxonomic upheaval (number of name changes). Adopting such a solution would first require clear understanding of the relationships of the bloodwood lineages to each other and to *Angophora*, informed by nuclear data, as well as the chloroplast data presented here. In the interim, and in the absence of other strongly contradictory evidence, we support continued recognition of both *Angophora* and *Corymbia* and the infrageneric groups of *Corymbia* as currently defined [6]. This is because there is support for most of these groups based on nrDNA data and morphology, and because name changes that are not soundly based, or might subsequently need revision in the face of stronger evidence, would cause major taxonomic instability in these economically important groups.

## Supporting information

S1 File Workflow used in CLC Genomics Workbench v. 9.5.1 and 9.5.2 (Qiagen). Quality control and assembly of HTS chloroplast DNA sequences (Excel file).
(XLSX)

S2 File Alignment of chloroplast data. Alignment (121,016 base pairs) of chloroplast sequences for 123 accessions of eucalypts (phylip file format).
(PHY)

S3 File Alignment of nuclear ribosomal data. Alignment (663 base pairs) of nrITS sequences for 119 accessions of eucalypts (phylip file format).
(PHY)

## References

[1] CHAH (2017) Australian plant census [online]. Council of Heads of Australasian Herbaria. Available from: https://biodiversity.org.au/nsl/services/apc.

[2] Hill KD, Johnson LA (1995) Systematic studies in the eucalypts 7. A revision of the bloodwoods, genus *Corymbia* (Myrtaceae). Telopea 6: 185–504.

[3] Pryor LD, Johnson LA (1971) A classification of the eucalypts Canberra: Australian National University Press.

[4] Brooker MIH (2000) A new classification of the genus *Eucalyptus* L'Her. (Myrtaceae). Aust Syst Bot 13: 79–148.

[5] Nicolle D (2015) Classification of the eucalypts. version 2. Available from: http://www.dn.com.au/Classification-Of-The-Eucalypts.pdf.

[6] Parra O C, Bayly MJ, Drinnan A, Udovicic F, Ladiges P (2009) Phylogeny, major clades and infrageneric classification of *Corymbia* (Myrtaceae), based on nuclear ribosomal DNA and morphology. Aust Syst Bot 22: 384–399.

[7] Williams JE, Woinarski JCZ (1997) Eucalypt Ecology: Individuals to Ecosystems. Cambridge Cambridge University Press.

[8] Chippendale GM (1988) Flora of Australia Vol. 19 Canberra: Australian Government Publishing Service.

[9] Boland DJ, Brooker MIH, Chippendale G, Hall N, Hyland B, et al (2006) Forest trees of Australia Melbourne: CSIRO publishing.

[10] MacPherson J (1939) The *Eucalyptus* in the Daily Life and Medical Practice of the Australian Aborigines. Mankind 2: 175–180.

[11] Packer J, Brouwer N, Harrington D, Gaikwad J, Heron R, et al (2012) An ethnobotanical study of medicinal plants used by the Yaegl Aboriginal community in northern New South Wales, Australia. J Ethnopharmacol 139: 244–255. doi: 10.1016/j.jep.2011.11.008 22101358 10.1016/j.jep.2011.11.008

[12] Reid EJ, Betts TJ (1979) Records of Western Australian Plants Used by Aboriginals as Medicinal Agents. Planta Med 36: 164–173. doi: 10.1055/s-0028-1097257 461569 10.1055/s-0028-1097257

[13] Batish DR, Singh HP, Kohli RK, Kaur S (2008) *Eucalyptus* essential oil as a natural pesticide. For Ecol Manage 256: 2166–2174.

[14] Low D, Rawal BD, Griffin WJ (1974) Antibacterial action of the essential oils of some Australian Myrtaceae with special references to the activity of chromatographic fractions of oil of *Eucalyptus citriodora*. Planta Med 26: 184–189. doi: 10.1055/s-0028-1097987 4212944 10.1055/s-0028-1097987

[15] Ramezani H, Singh HP, Batish DR, Kohli RK (2002) Antifungal activity of the volatile oil of *Eucalyptus citriodora*. Fitoterapia 73: 261–262. 12048022 10.1016/s0367-326x(02)00065-5

[16] Ladiges PY, Udovicic F, Nelson G (2003) Australian biogeographical connections and the phylogeny of large genera in the plant family Myrtaceae. J Biogeogr 30: 989–998.

[17] Wilson PG, O'Brien M, Heslewood M, Quinn C (2005) Relationships within Myrtaceae sensu lato based on a matK phylogeny. Pl Syst Evol 251: 3–19.

[18] Slee AV, Connors J, Brooker MIH, Duffy SM, West JG (2006) EUCLID Eucalypts of Australia. CD ROM Centre for Plant Biodiversity Research. Melbourne: CSIRO Publishing.

[19] Dawson JW (1970) Pacific capsular Myrtaceae 1. Reproductive morphology of *Arillastrum gummiferum* Panch. ex Baillon (New Caledonia). Blumea 18: 431–440.

[20] Carr DJ, Carr S, Hyland B, Wilson PG, Ladiges PY (2002) *Stockwellia quadrifida* (Myrtaceae), a new Australian genus and species in the eucalypt group. Bot J Linn Soc 139: 415–421.

[21] Blake S (1977) *Allosyncarpia ternata*, a new genus and species of Myrtaceae subfamily Leptospermoideae from northern Australia. Austrobaileya: 43–46.

[22] Craven L (1990) One new species each in *Acmena* and *Eucalyptopsis* and a new name in *Lindsayomyrtus* (all Myrtaceae). Aust Syst Bot 3: 727–732.

[23] Ladiges PY, Udovicic F (2000) Comment on a new classification of the eucalypts. Aust Syst Bot 13: 149–152.

[24] Ladiges PY, Udovicic F, Drinnan AN (1995) Eucalypt phylogeny–molecules and morphology. Aust Syst Bot 8: 483–497.

[25] Udovicic F, McFadden G, Ladiges P (1995) Phylogeny of *Eucalyptus* and *Angophora* based on 5S rDNA spacer sequence data. Mol Phyl Evol 4: 247–256. 10.1006/mpev.1995.1023 8845962

[26] Udovicic F, Ladiges PY (2000) Informativeness of nuclear and chloroplast DNA relationships of the eucalypt and related genera (Myrtaceae). Kew Bull 55: 633–645.

[27] Parra O C, Bayly MJ, Udovicic F, Ladiges PY (2006) ETS sequences support the monophyly of the eucalypt genus *Corymbia* (Myrtaceae). Taxon 55: 653–663.

[28] Whittock S, Steane D, Vaillancourt R, Potts B (2003) Molecular evidence shows that the tropical boxes (*Eucalyptus* subgenus *Minutifructus*) are over-ranked. Trans R Soc S Aust 1217: 27–32.

[29] Steane DA, McKinnon GE, Vaillancourt RE, Potts BM (1999) ITS sequence data resolve higher level relationships among the eucalypts. Mol Phyl Evol 12: 215–223. 10.1006/mpev.1999.0612 10381324

[30] Steane DA, Nicolle D, McKinnon GE, Vaillancourt RE, Potts BM (2002) Higher-level relationships among the eucalypts are resolved by ITS-sequence data. Aust Syst Bot 15: 49–62.

[31] González-Orozco CE, Pollock LJ, Thornhill AH, Mishler BD, Knerr N, et al (2016) Phylogenetic approaches reveal biodiversity threats under climate change. Nat Clim Change 6: 1110–1114.

[32] Ladiges P (1984) A comparative study of Trichomes in *Angophora* Cav. and *Eucalyptus*–a question of homology. Aust J Bot 32: 561–574.

[33] Bentham G (1866) Flora Australiensis, III. London: Lovell Reeve.

[34] Maiden JH (1909) A critical revision of the genus *Eucalyptus* Sydney: Gullick.

[35] Blakely WF (1965) A Key to the Eucalypts Canberra: Forestry and Timber Bureau.

[36] Cavanilles A (1797) Icones et Descriptiones Plantarum, IV. Madrid: Regia Typographia.

[37] Bayly MJ (2016) Phylogenetic studies of eucalypts: fossils, morphology and genomes. Proc R Soc Vic 128: 12–24.

[38] Ochieng JW, Henry RJ, Baverstock PR, Steane DA, Shepherd M (2007) Nuclear ribosomal pseudogenes resolve a corroborated monophyly of the eucalypt genus *Corymbia* despite misleading hypotheses at functional ITS paralogs. Mol Phyl Evol 44: 752–764. 10.1016/j.ympev.2007.04.017 17570687

[39] Ochieng JW, Steane DA, Ladiges PY, Baverstock PR, Henry RJ, et al (2007) Microsatellites retain phylogenetic signals across genera in eucalypts (Myrtaceae). Genet Mol Biol 30: 1125–1134.

[40] Parra-O C (2009) Chapter 6, Chloroplast DNA (cpDNA). In: Parra-O C, editor. A phylogenetic analysis of the bloodwood eucalypts (Myrtaceae): PhD Thesis, The University of Melbourne.

[41] Bayly MJ, Rigault P, Spokevicius A, Ladiges PY, Ades PK, et al (2013) Chloroplast genome analysis of Australian eucalypts–*Eucalyptus*, *Corymbia*, *Angophora*, *Allosyncarpia* and *Stockwellia* (Myrtaceae). Mol Phyl Evol 69: 704–716. 10.1016/j.ympev.2013.07.006 23876290

[42] Steane DA (2005) Complete nucleotide sequence of the chloroplast genome from the Tasmanian blue gum, *Eucalyptus globulus* (Myrtaceae). DNA Res 12: 215–220. doi: 10.1093/dnares/dsi006 16303753 10.1093/dnares/dsi006

[43] Paiva JA, Prat E, Vautrin S, Santos MD, San-Clemente H, et al (2011) Advancing *Eucalyptus* genomics: identification and sequencing of lignin biosynthesis genes from deep-coverage BAC libraries. BMC Genomics 12: 137 doi: 10.1186/1471-2164-12-137 21375742 10.1186/1471-2164-12-137 PMC3060884

[44] Doyle JJ, Doyle JL (1987) A rapid isolation procedure for small quantities of fresh leaf material. Phytochem Bull 19: 11–15.

[45] McLay TG (2017) High quality DNA extraction protocol from recalcitrant plant tissues. protocols.io. Available from: https://www.protocols.io/view/high-quality-dna-extraction-protocol-from-recalcit-i8jchun?more.

[46] Shepherd LD, McLay TG (2011) Two micro-scale protocols for the isolation of DNA from polysaccharide-rich plant tissue. J Plant Res 124: 311–314. doi: 10.1007/s10265-010-0379-5 20927638 10.1007/s10265-010-0379-5

[47] Rohland N, Reich D (2012) Cost-effective, high-throughput DNA sequencing libraries for multiplexed target capture. Genome Res 22: 939–946. doi: 10.1101/gr.128124.111 22267522 10.1101/gr.128124.111 PMC3337438

[48] Katoh K, Standley DM (2013) MAFFT multiple sequence alignment software version 7: improvements in performance and usability. Mol Biol Evol 30: 772–780. doi: 10.1093/molbev/mst010 23329690 10.1093/molbev/mst010 PMC3603318

[49] Gouy M, Guindon S, Gascuel O (2009) SeaView version 4: a multiplatform graphical user interface for sequence alignment and phylogenetic tree building. Mol Biol Evol 27: 221–224. doi: 10.1093/molbev/msp259 19854763 10.1093/molbev/msp259

[50] Maddison WP, Maddison DR (2013) Mesquite version 3.1: a modular system for evolutionary analysis. http://mequiteproject.org/.

[51] Castresana J (2000) Selection of conserved blocks from multiple alignments for their use in phylogenetic analysis. Mol Biol Evol 17: 540–552. doi: 10.1093/oxfordjournals.molbev.a026334 10742046 10.1093/oxfordjournals.molbev.a026334

[52] Darriba D, Taboada GL, Doallo R, Posada D (2012) jModelTest 2: more models, new heuristics and parallel computing. Nat Methods 9: 772–772. 10.1038/nmeth.2109 PMC4594756 22847109

[53] Guindon S, Gascuel O (2003) A simple, fast, and accurate algorithm to estimate large phylogenies by maximum likelihood. Syst Biol 52: 696–704. 14530136 10.1080/10635150390235520

[54] Stamatakis A (2014) RAxML version 8: a tool for phylogenetic analysis and post-analysis of large phylogenies. Bioinformatics 30: 1312–1313. doi: 10.1093/bioinformatics/btu033 24451623 10.1093/bioinformatics/btu033 PMC3998144

[55] Swofford D (2002) PAUP*. Phylogenetic Analysis Using Parsimony (* and Other Methods). 4 ed: Sinauer Associates, Sunderland, Massachusetts.

[56] Rambaut A (2016) FigTree Tree Figure Drawing Tool version 1.4.3. http://tree.bio.ed.ac.uk/software/figtree/.

[57] Gibbs AK, Udovicic F, Drinnan AN, Ladiges PY (2009) Phylogeny and classification of *Eucalyptus* subgenus *Eudesmia* (Myrtaceae) based on nuclear ribosomal DNA, chloroplast DNA and morphology. Aust Syst Bot 22: 158–179.

[58] Ladiges PY, Bayly M, Nelson G (2010) East-west continental vicariance in *Eucalyptus* subgenus *Eucalyptus* In: Williams D, Knapp S, editors. Beyond Cladistics. The Branching of a Paradigm. Berkeley: University of California Press pp. 267–302.

[59] Bayly MJ, Ladiges PY (2007) Divergent paralogues of ribosomal DNA in eucalypts (Myrtaceae). Mol Phyl Evol 44: 346–356. 10.1016/j.ympev.2006.10.027 17188000

[60] Bayly MJ, Udovicic F, Gibbs AK, Ladiges PY (2008) Ribosomal DNA pseudogenes are widespread in the eucalypt group (Myrtaceae): implications for phylogenetic analysis. Cladistics 24: 131–146.

[61] Burke JM, Bayly MJ, Adams PB, Ladiges PY (2008) Molecular phylogenetic analysis of *Dendrobium* (Orchidaceae), with emphasis on the Australian section *Dendrocoryne*, and implications for generic classification. Aust Syst Bot 21: 1–14.

[62] Holmes GD, Downing TL, James EA, Blacket MJ, Hoffmann AA, et al (2014) Phylogeny of the holly grevilleas (Proteaceae) based on nuclear ribosomal and chloroplast DNA. Aust Syst Bot 27: 56–77.

[63] Kearse M, Moir R, Wilson A, Stones-Havas S, Cheung M, et al (2012) Geneious Basic: an integrated and extendable desktop software platform for the organization and analysis of sequence data. Bioinformatics 28: 1647–1649. doi: 10.1093/bioinformatics/bts199 22543367 10.1093/bioinformatics/bts199 PMC3371832

[64] Bailey CD, Carr TG, Harris SA, Hughes CE (2003) Characterization of angiosperm nrDNA polymorphism, paralogy, and pseudogenes. Mol Phyl Evol 29: 435–455. 10.1016/j.ympev.2003.08.021 14615185

[65] Mayol M, Rosselló JA (2001) Why nuclear ribosomal DNA spacers (ITS) tell different stories in *Quercus*. Mol Phyl Evol 19: 167–176. 10.1006/mpev.2001.0934 11341800

[66] Baum DA, Smith SD (2013) Tree thinking: an introduction to phylogenetic biology. Greenwood Village, Colarado: Roberts and Company.

[67] Doyle JJ (1992) Gene trees and species trees: molecular systematics as one-character taxonomy. Syst Bot: 144–163.

[68] Maddison WP (1997) Gene trees in species trees. Syst Biol 46: 523–536.

[69] Tsitrone A, Kirkpatrick M, Levin DA, Morgan M (2003) A model for chloroplast capture. Evolution 57: 1776–1782. 14503619 10.1111/j.0014-3820.2003.tb00585.x

[70] Rieseberg LH, Soltis D (1991) Phylogenetic consequences of cytoplasmic gene flow in plants. Evol Trends Pl 5: 65–84.

[71] McKinnon GE, Jordan GJ, Vaillancourt RE, Steane DA, Potts BM (2004) Glacial refugia and reticulate evolution: the case of the Tasmanian eucalypts. Philos Trans R Soc Lond, B, Biol Sci 359: 275–284. doi: 10.1098/rstb.2003.1391 15101583 10.1098/rstb.2003.1391 PMC1693314

[72] Meudt HM, Bayly MJ (2008) Phylogeographic patterns in the Australasian genus *Chionohebe* (*Veronica* s.l., Plantaginaceae) based on AFLP and chloroplast DNA sequences. Mol Phyl Evol 47: 319–338. 10.1016/j.ympev.2007.12.019 18299210

[73] Dickinson GR, Lee DJ, Wallace HM (2012) The influence of pre-and post-zygotic barriers on interspecific *Corymbia* hybridization. Ann Bot 109: 1215–1226. doi: 10.1093/aob/mcs050 22419764 10.1093/aob/mcs050 PMC3359913

[74] Grattapaglia D, Vaillancourt RE, Shepherd M, Thumma BR, Foley W, et al (2012) Progress in Myrtaceae genetics and genomics: *Eucalyptus* as the pivotal genus. Tree Genetics & Genomes 8: 463–508.

[75] Oudjehih B, Abdellah B (2006) Chromosome numbers of the 59 species of *Eucalyptus* L'Herit. (Myrtaceae). Caryologia 59: 207–212.

[76] Barbour R, Crawford AC, Henson M, Lee DJ, Potts B, et al (2008) The risk of pollen-mediated gene flow from exotic *Corymbia* plantations into native *Corymbia* populations in Australia. For Ecol Manage 256: 1–19.

[77] Griffin A, Burgess I, Wolf L (1988) Patterns of natural and manipulated hybridisation in the genus *Eucalyptus* L’Hér.–a review. Aust J Bot 36: 41–66.

[78] Jackson H, Steane D, Potts B, Vaillancourt R (1999) Chloroplast DNA evidence for reticulate evolution in *Eucalyptus* (Myrtaceae). Mol Ecol 8: 739–751.

[79] McKinnon G, Smith J, Potts B (2010) Recurrent nuclear DNA introgression accompanies chloroplast DNA exchange between two eucalypt species. Mol Ecol 19: 1367–1380. doi: 10.1111/j.1365-294X.2010.04579.x 20298471 10.1111/j.1365-294X.2010.04579.x

[80] McKinnon GE, Steane DA, Potts BM, Vaillancourt RE (1999) Incongruence between chloroplast and species phylogenies in *Eucalyptus* subgenus *Monocalyptus* (Myrtaceae). Am J Bot 86: 1038–1046. 10406727

[81] McKinnon GE, Vaillancourt RE, Jackson HD, Potts BM (2001) Chloroplast sharing in the Tasmanian eucalypts. Evolution 55: 703–711. 11392388 10.1554/0014-3820(2001)055[0703:csitte]2.0.co;2

[82] Steane D, Byrne M, Vaillancourt R, Potts B (1998) Chloroplast DNA polymorphism signals complex interspecific interactions in *Eucalyptus* (Myrtaceae). Aust Syst Bot 11: 25–40.

[83] Nevill PG, Després T, Bayly MJ, Bossinger G, Ades PK (2014) Shared phylogeographic patterns and widespread chloroplast haplotype sharing in *Eucalyptus* species with different ecological tolerances. Tree Genetics & Genomes 10: 1079–1092.

[84] Pollock LJ, Bayly MJ, Nevill PG, Vesk PA (2013) Chloroplast DNA diversity associated with protected slopes and valleys for hybridizing *Eucalyptus* species on isolated ranges in south-eastern Australia. J Biogeogr 40: 155–167.

[85] Pollock LJ, Bayly MJ, Vesk PA (2015) The roles of ecological and evolutionary processes in plant community assembly: the environment, hybridization, and introgression influence co-occurrence of *Eucalyptus*. Am Nat 185: 784–796. doi: 10.1086/680983 25996863 10.1086/680983

[86] Leach GJ (1986) A revision of the genus *Angophora* (Myrtaceae). Telopea 2: 749–779.

[87] Thiele K, Ladiges PY (1988) A cladistic analysis of *Angophora* Cav. (Myrtaceae). Cladistics 4: 23–42. 10.1111/j.1096-0031.1988.tb00466.x 34933494

